# ProKinO: An Ontology for Integrative Analysis of Protein Kinases in Cancer

**DOI:** 10.1371/journal.pone.0028782

**Published:** 2011-12-14

**Authors:** Gurinder Gosal, Krys J. Kochut, Natarajan Kannan

**Affiliations:** 1 Department of Biochemistry and Molecular Biology, University of Georgia, Athens, United States of America; 2 Department of Computer Science, University of Georgia, Athens, United States of America; 3 Institute of Bioinformatics, University of Georgia, Athens, United States of America; Wayne State University School of Medicine, United States of America

## Abstract

**Background:**

Protein kinases are a large and diverse family of enzymes that are genomically altered in many human cancers. Targeted cancer genome sequencing efforts have unveiled the mutational profiles of protein kinase genes from many different cancer types. While mutational data on protein kinases is currently catalogued in various databases, integration of mutation data with other forms of data on protein kinases such as sequence, structure, function and pathway is necessary to identify and characterize key cancer causing mutations. Integrative analysis of protein kinase data, however, is a challenge because of the disparate nature of protein kinase data sources and data formats.

**Results:**

Here, we describe ProKinO, a protein kinase-specific ontology, which provides a controlled vocabulary of terms, their hierarchy, and relationships unifying sequence, structure, function, mutation and pathway information on protein kinases. The conceptual representation of such diverse forms of information in one place not only allows rapid discovery of significant information related to a specific protein kinase, but also enables large-scale integrative analysis of protein kinase data in ways not possible through other kinase-specific resources. We have performed several integrative analyses of ProKinO data and, as an example, found that a large number of somatic mutations (∼288 distinct mutations) associated with the *haematopoietic neoplasm* cancer type map to only 8 kinases in the human kinome. This is in contrast to *glioma*, where the mutations are spread over 82 distinct kinases. We also provide examples of how ontology-based data analysis can be used to generate testable hypotheses regarding cancer mutations.

**Conclusion:**

We present an integrated framework for large-scale integrative analysis of protein kinase data. Navigation and analysis of ontology data can be performed using the ontology browser available at: http://vulcan.cs.uga.edu/prokino.

## Introduction

Cancer is caused by an accumulation of mutations, often in a subset of genes that confer survival and growth advantage. The protein kinase gene family, which controls key signaling pathways associated with cell growth and survival, is one of the most over-represented families of oncogenes [1]. Targeted sequencing of 518 protein kinase exons encoded in the human genome (collectively called the kinome) has revealed hundreds of mutations in the protein kinase domain [2]. Although these mutations are currently catalogued in various databases [3], [4], [5], identification and experimental characterization of key cancer-causing mutations is essential for developing new therapies for cancer.

Experimental characterization of cancer mutations, however, requires that one first formulate the right hypotheses based on analysis of existing data. In particular, analysis of mutation data in light of other forms of data available on protein kinases such as sequence, structure, function and pathway is necessary to develop and test new hypotheses regarding the functional impact of cancer mutations [6], [7], [8], [9]. Integrative analysis of protein kinase data, however, is a challenge because of the disparate nature of protein kinase data sources and formats. For example, a researcher interested in the structural location of a cancer mutation, or distribution of kinase mutations in various cancer types, has to go through the time-consuming and error prone process of collecting and parsing data from disparate sources, often in different data formats. Although several kinase-specific resources such as KinBase [10], KING [11], PKR [12] and KinMutBase [4] have been developed, these resources largely focus on one, or few types, of protein kinase data (e.g., sequence, structure, or mutation), leaving aside the challenge of data integration.

Ontologies [13] have emerged as a powerful tool for integrative and quantitative analysis of biological data [14], [15], [16], [17]. By capturing domain knowledge in the form of concepts (classes) and relationships, ontologies provide a conceptual representation of data in a way that computers can read and humans can understand. For example, for an automated and informed response to the query “kinase mutations associated with cancer types”, the computer needs to understand the concepts, “kinase mutations” and “cancer types”, and the relationships between the concepts, namely, “*associated with”*. It is this conceptual representation of knowledge that distinguishes ontologies from relational databases, and enables efficient integration and mining of diverse data sets [18]. Indeed, several ontologies have been developed to capture and mine the wealth of information on genes (GO) [19], sequence [20], pathways (http://rgd.mcw.edu/tools/ontology/ont_search.cgi *)*, protein modification [21] and others [20], [22]. Focused ontologies on selected protein families such as the protein phosphatase family and transporter family have also been developed [23]. However, up until now, a focused ontology capturing the state of knowledge on the protein kinase family has not been reported.

Here, we report the Protein Kinase Ontology (ProKinO). ProKinO provides a controlled vocabulary of terms and relationships connecting sequence, structure, function, pathway, and mutation data on protein kinases. ProKinO is encoded using the Web Ontology Language (OWL) (http://www.w3.org/TR/owl-ref/), an ontology authoring language recommended by the World Wide Web Consortium (http://www.w3.org/). The integration of diverse data sets in a machine-readable format not only allows navigation of diverse forms of protein kinase data in one place, but also enables aggregate queries on existing data in ways not possible through existing kinase-specific resources. For example, aggregate queries such as “counts of kinases associated with cancer type” or “counts of cancer mutations located in various kinase sub-domains” can be readily performed using ProKinO and the ontology query language SPARQL (http://www.w3.org/TR/rdf-sparql-query/). We describe the significance of such queries in knowledge discovery and hypothesis generation. An aggregate query “counts of kinase mutations in various cancer types”, for example, revealed that the mutations associated with *haematopoietic neoplasm* (288 distinct mutations) primarily target only 8 kinases in the human kinome, compared to *glioma*, where the mutations are spread over 82 distinct kinases. Likewise, queries such as “mutations targeting kinase functional features” can be used to generate new hypotheses regarding the structural and functional impact of cancer mutations. We also describe a browser that enables rapid navigation and examination of ProKinO data, accessible at: http://vulcan.cs.uga.edu/prokino.

## Methods

### ProKinO Knowledge Organization

To conceptualize the wealth of knowledge regarding protein kinase sequence, structure, function, pathways and diseases, we have introduced several key concepts (classes) and relationships (object properties) in ProKinO. These classes, organized in a hierarchical manner, and the relationships amongst these classes, represent and describe protein kinase knowledge in a manner analogous to a domain expert.

**Figure 1:**
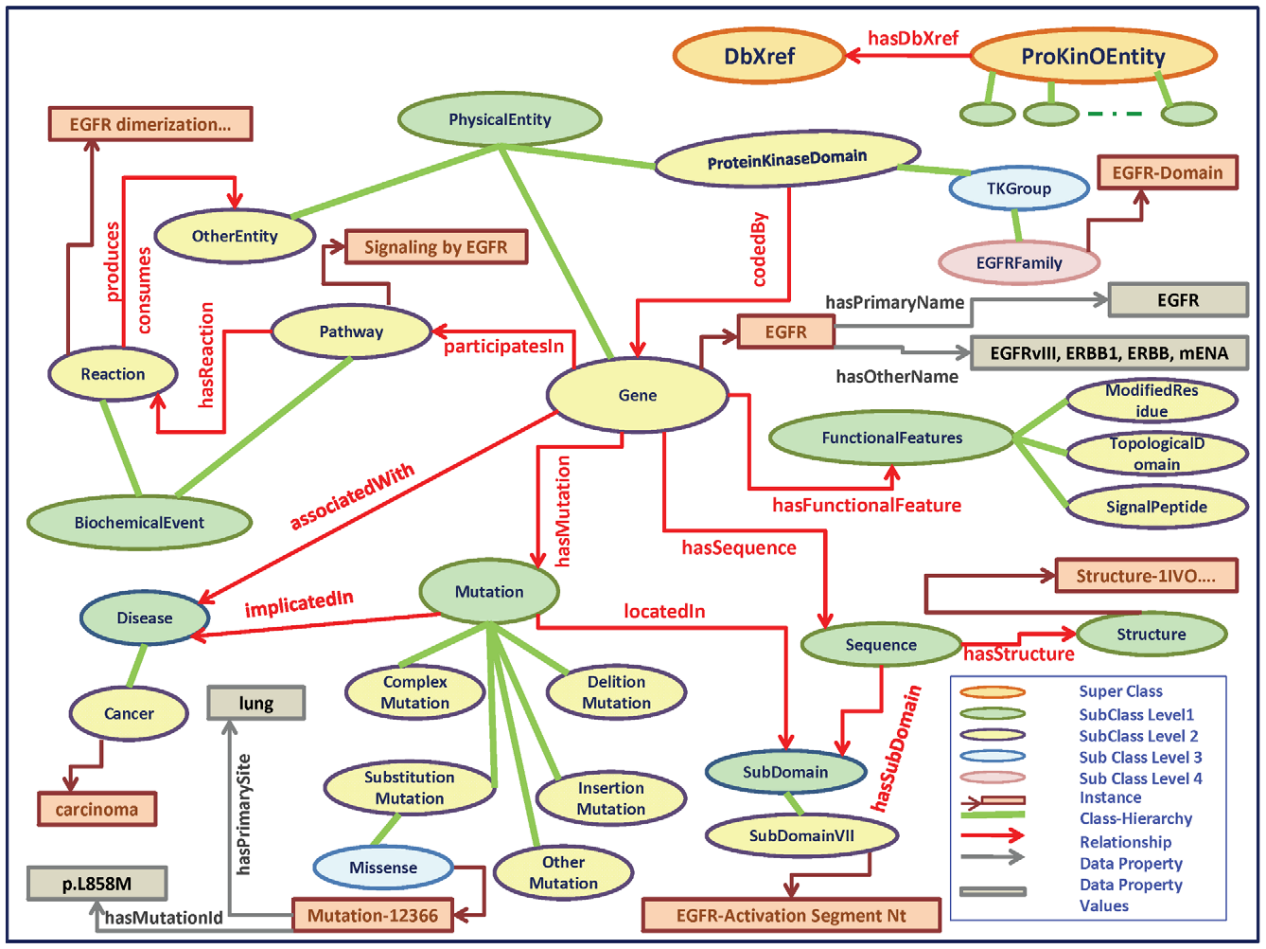
A section of the Protein Kinase Ontology (ProKinO) schema showing key concepts and relationships. The figure shows concepts (classes) organized in a class sub-class hierarchy (shown as ovals). The relationships (object properties) between classes are shown as red colored lines. The internal specifics (data properties) of classes are shown as brown colored lines. The instances of classes are shown as rectangles. The complete ontology schema can be accessed from the ProKinO web site, and also provided as Figure S1.

For example, a kinase expert describing a particular mutation would describe the mutation in the context of the gene in which the mutation is found, the kinase encoded by the gene, the group or family the kinase belongs to, the kinase sub-domain the mutation is located in, and the pathways in which the mutated gene participates. The ProKinO schema has been designed to capture and integrate protein kinase knowledge using the terms and relationships similar to those typically used by an expert (Figure 1). For example, the relationship between the “Gene” and “Mutation” classes is described by the “*hasMutation”* property (Figure 1), while the “*locatedIn”* property captures the relationship between the “Mutation” and “SubDomain” classes. Similarly, the sequence a kinase belongs to is represented by the “*hasSequence”* property between the “Gene” and “Sequence“ classes, and the sub-domains associated with a particular sequence is conceptualized by the “*hasSubDomain”* relationship (Figure 1). The pathway and reaction information related to kinases is conceptualized by the “*participatesIn”* relationship between “Gene” and “Pathway”, and “*hasReaction”* between “Pathway” and “Reaction”. To cross reference ProKinO data to external databases and sources, the “DbXref” class and “*hasDbXref”* relationship have been introduced (see Figure 1).

The rationale behind representing protein kinase data in the above described way is that it provides context for interpreting mutation data. This can be illustrated using the missense mutation *p.L858M* in *EGFR* (Figure 1). *p.L858M* is a mutation in *EGFR* kinase having the type “Missense”. The mutation is implicated in cancer *carcinoma* and located in the sub domain VII, which corresponds to the N-terminus of the Activation segment (denoted as *Activation-Segment-NT* in Figure 1). The protein encoded by the *EGFR* gene participates in a pathway *Signaling by EGFR*, which includes *EGFR dimerization* as one of its reactions. Other classes and sub-classes are likewise connected to the mutation *p.L858M* via the relationships described in Figure 1, providing an integrated view of all data that would be required to provide structural and functional context for the *p. L858M* mutation.

In addition to the major classes and object properties described above, several additional sub-classes and object properties have been defined in ProKinO to fully capture and represent the available knowledge on protein kinase sequence, structure, function and disease. For example, the sub-classes of the “Mutation” class — “ComplexMutation”, “DeletionMutation”, “InsertionMutation”, “SubstitutionMutation” and “OtherMutation” — capture information on the types of mutations identified in kinases. Likewise, the three sub-classes under the “FunctionalFeature” class — “ModifiedResidue”, “TopologicalDomain”, “SignalPeptide” — capture information on the specific functional features. This hierarchal organization of classes in ProKinO is shown in Figure 1.

In addition to the object properties, key data properties have been introduced to describe the internal organization of the concepts and to facilitate data mining and extraction. For example, the data property, “*hasOtherName”*, stores the other names by which a gene may be known in the literature (synonyms). For instance, *EGFR* is also referred as *EGFRvIII*, *ERBB1*, *ERBB*, or *mENA* in the literature. By including the “*hasOtherName”* data property, all information pertinent to *EGFR* can be obtained irrespective of which gene name is used as a query.

With a large set of classes and properties related to kinases in the designed schema (refer to Figure S1 for the full schema), ProKinO, represents an explicit conceptualization and organization of the knowledge about human protein kinases. ProKinO currently contains 351 classes, 25 object properties and 27 data properties (Tables S1, S2 and S3 for full list) capturing information on protein kinase sequence, structure, function, pathway and disease.

### ProKinO Population

ProKinO has been populated with data from data sources that are well curated and maintained. The acquired data has been stored as instances in the schema described above (Figure 1).

### Data acquisition and storage

#### Sequence

Data regarding protein kinase sequence and classification have been obtained from KinBase [10], the repository for kinase sequence and classification. The 538 kinase genes currently identified in the human genome have been classified into major groups and families based on sequence similarity within the kinase domain. Since the KinBase classification is widely accepted by the kinase community, we have adopted the same classification scheme in ProKinO. The automatic process of data acquisition and population from KinBase includes the extraction, integration and population of information from 538 human protein kinases and their classification into various groups, families and subfamilies. Information regarding gene names, synonyms and chromosomal position is also obtained from KinBase. The acquired knowledge is populated as the instances of the “ProteinKinaseDomain” class, which is further categorized into groups, families, and sub-families as subclasses. Further, the sequence data of protein kinase genes in FASTA format has been extracted and populated as instances of the “Sequence” class.

#### Function

Information regarding functional domains and functional features associated with kinase domains have been obtained from UniProt [24], a curated resource for protein functional information. Information on the regulatory domains associated with kinase domains, crystal structures solved for each kinase, isoforms identified for kinases, modified residue, signal peptide, topological domain, cellular location and tissue specificity is also obtained from UniProt. Functional domains related to protein kinases are populated as instances of the “FunctionalDomain” class, and cross referenced to Pfam [25], a protein family database, via the “DBxRef” class. Similarly, information about crystal structures is populated as instances of the “Structure” class with cross references to the Protein Data Bank (PDB) [26]. Functional feature information is stored as instances in the “FunctionalFeature” class, with sub-classes based on the type of feature such as “ModifiedResidue”, “TopologicalDomain” and “SignalPeptide”.

#### Disease

Although protein kinases have been associated with several human diseases, the current version of ProKinO primarily focuses on cancer. Information regarding cancer mutations is obtained from COSMIC [3], which is one of the oldest and curated resources for storing information on somatic acquired mutations associated with human cancers. In addition to mutations, other information such as primary sites, primary histology, samples, description and other relevant features have also been obtained and stored as instances in the “Mutation” class. The “Mutation” class is specialized further into sub-classes based on the type of mutation, namely, complex, deletion, insertion, substitution and other. References to PubMed, MEDLINE and COSMIC databases are provided in the “DbXref” class.

#### Pathway

Pathway data is obtained from Reactome, a manually curated and peer-reviewed pathway resource [27]. Pathways and reaction are stored as instances in the “BiochemicalEvent” class. For the sake of clarity, we have adopted the same terms/concepts used in Reactome to represent pathway information. “BiochemicalEvent” is a concept used in both Reactome and ProKinO to represent biological processes that convert input entities to output entities. “Pathway” and “Reaction” are sub-classes under “BiochemicalEvent” (Figure 1). For example, *Signaling by EGFR* is an instance in the “Pathway” class, which is related to the “Reaction” class by the “*hasReaction”* property (Figure 1). The “Reaction” class has several reactions for a given pathway. *EGFR dimerization* is one of the reactions in the *Signaling by EGFR* pathway (Figure 1). This reaction “*consumes”* a complex named *EGF:EGFR [plasma membrane]*, and “*produces”* a complex, *EGF:EGFR dimer [plasma membrane]*. Both complexes are stored as members of the “Complex” class.

#### Kinase Sub-domains

To provide structural context for cancer mutations, we have incorporated sub-domain information in ProKinO. Sub-domains correspond to the core conserved motifs/structural elements that define the kinase catalytic domain [28]. The sub-domain notation is widely used to describe the structural organization of motifs and regulatory segments that make up the catalytic domain. Currently, sub-domain information on human kinases is not available from any public resource. The protein kinase resource (PKR) provides sub-domain information on some (18 kinases), but not on all kinases. To capture the sub-domain information in ProKinO, we have used a motif model, which captures key motifs corresponding to each of XII sub-domains in the kinase domain [6], [29]. The motif model was run against all UniProt and COSMIC sequences to identify the start and end location of sub-domains in sequences. The start and end locations of sub-domains have been stored in ProKinO as instances in the “SubDomain” class. Because sub-domain boundaries are difficult to delineate for divergent protein kinases, such as the atypical kinases, the sub-domain class is not populated for all protein kinases.

### Automation of data acquisition and updates

We have created a specialized software system to automatically populate ProKinO from the above described sources. The software is written using the Java programming language. The software performs all of the required functions for ontology creation and automatic population, including data acquisition, parsing and processing, as well as the creation of instances and connections among them using the relationships defined in the ProKinO schema. The populated ontology is encoded and output in OWL, an ontology authoring and sharing language recommended by the World Wide Web Consortium. Our software also uses Jena, a widely used Java-based Application Programming Interface (API) (http://jena.sourceforge.net/) for parsing, creating and querying Resource Description Framework (RDF) (http://www.w3.org/RDF/ *)* and OWL ontologies.

The ontologies, and hence any software applications and resources utilizing them, are bound to evolve with time. ProKinO integrates knowledge from disparate sources without modifying any of the original data. Therefore, any changes in the data sources used in ProKinO creation require the corresponding changes in the ontology to assure that it is up-to-date and consistent. The sources of knowledge used in ProKinO are subject to frequent modifications and are updated on a regular basis. For instance, UniProt is updated every three weeks and COSMIC approximately every two months. For the knowledge integrated in the ontology to be current and consistent with the existing data available in the parent sources, ProKinO will be updated by our automatic population process on a regular basis, as well. The version information about all data sources used to populate ProKinO will be included, as well. To assure that the needs of user community are satisfied, any needed schema modifications and extensions will be introduced in new ProKinO versions at appropriate times. All of the versions of ProKinO will be archived along with the information about differences between versions. The ontology lifecycle will be tracked by a versioning system [30], and any prior versions of ProKinO will be easily accessible.

## Results and Discussion

### ProKinO Evaluation

Because the ontology development process is costly and time consuming, careful evaluation of ontology content is necessary to determine its suitability in serving the intended purpose of its development. ProKinO has been evaluated for its accuracy and usefulness. We have used two approaches to evaluate the accuracy of ProKinO content: (i) a manual approach in which a set of instances and relationships among them are randomly selected and cross-checked with content from original sources, and (ii) a query-based approach in which ontology data is queried for information that can easily be cross validated with data from original sources.

#### Manual Approach

In the manual approach, the test sets were chosen to evaluate a broad coverage of the ontology content. The accuracy of the data was checked by cross validating with the original data sources. The integration of the data in ProKinO was also verified by evaluating the introduced object and data properties for accuracy. For example, *EGFR* kinase's relationship with pathways represented as a property “*partcipatesIn”* was verified for accuracy by cross validating the content in ProKinO with the original data available in Reactome. Our verification has not detected any errors in ProKinO. The details of the evaluation are shown in Table S4.

#### Query-based Approach

**Figure 2:**
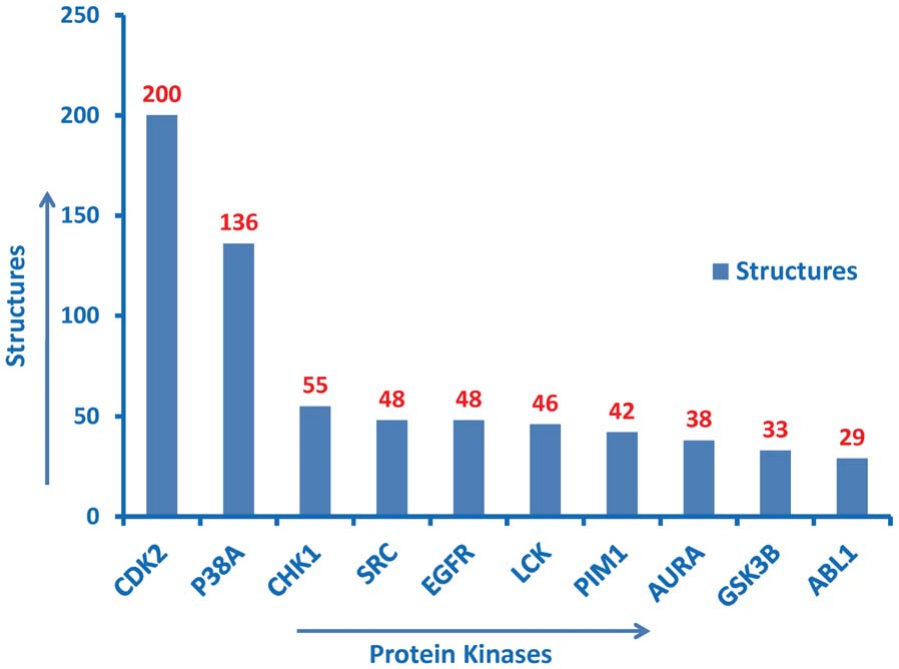
Counts of crystal structures of all protein kinases. Top ten kinases in the descending order of counts are displayed. The Y-axis shows the number of structures solved for each of ten over-represented kinases. Structures solved with inhibitors were included in the total count. X-axis denotes the kinase names. Aurora kinase is labeled as AURA. The SPARQL query used to generate this figure can be viewed and excuted from the ProKinO browser by selecting “Query 1” under the “Example queries” tab in the main page.

**Figure 3:**
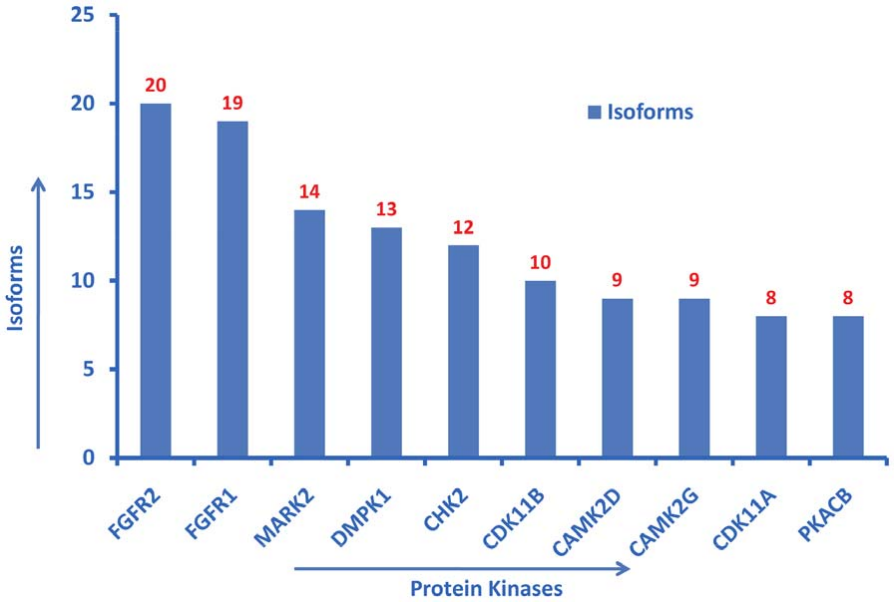
Counts of isoforms for all protein kinases. Top 10 kinases are displayed in descending order of their values. The Y-axis shows the number of validated isoforms for each of the kinass. The SPARQL query used to generate this figure can be viewed and excuted from the ProKinO browser by selecting “Query 2” under the “Example queries” tab in the main page.

**Figure 4:**
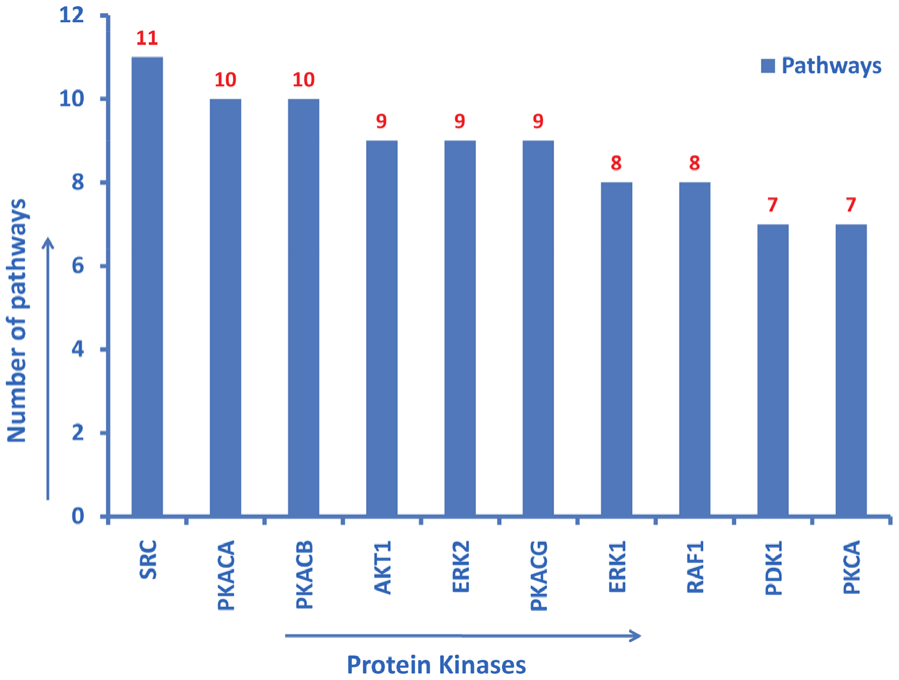
Counts of number of pathways associated with all protein kinases. Top 10 kinases with the most number of pathways are displayed in descending order. The SPARQL query to generate this figure can be directly viewed and excuted from the ProKinO browser by selecting “Query 3” under the “Example queries” tab in the main page.

**Figure 5:**
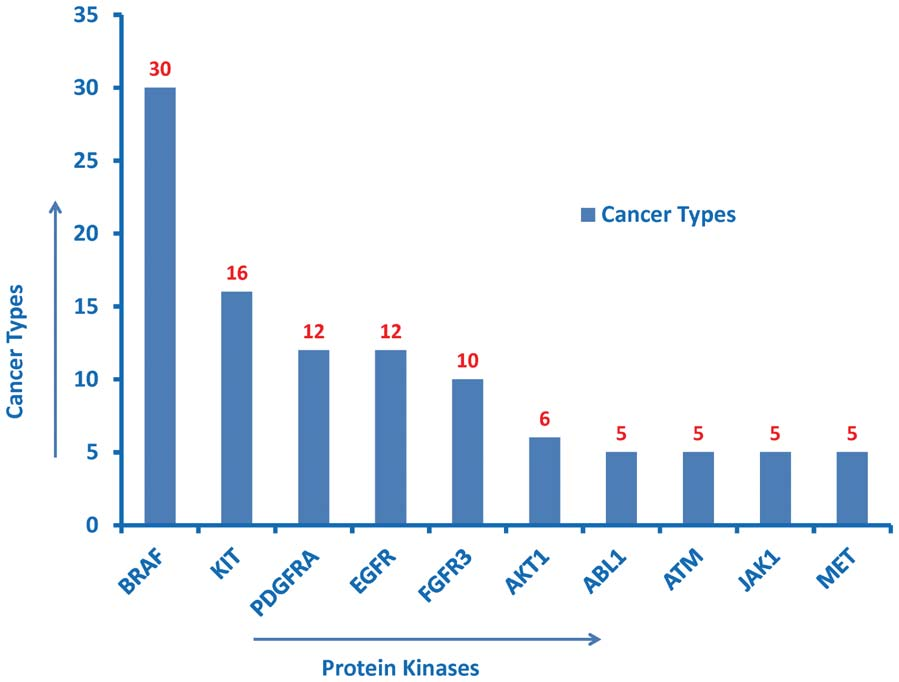
Counts of different cancer types implicated in protein kinases. Top ten kinases are in descending order of their values. The SPARQL query to generate this figure can be directly viewed and excuted from the ProKinO browser by selecting “Query 4” under the “Example queries” tab in the main page.

In addition to the manual approach, a query-based approach was used to verify the content of the ontology. The SPARQL query language was used to perform the queries. For example, the query “count of crystal structures for all protein kinases” resulted in 200 hits for *Cdk2* (Figure 2). This result was cross-validated by checking the *Cdk2* “PDB” entry in UniProt. Similarly, the query “count of isoforms for all protein kinases” resulted in 20 hits for *FGFR2* and 19 for *FGFR1* (Figure 3). This was cross-validated by checking for *FGFR1* and *FGFR2* isoform entries in UniProt. Likewise, “counts of kinases associated with pathways” resulted in 11 pathways for *SRC*, and 10 for *PKACA* (*PRKACA* in Reactome). This result was also cross-validated with the original source, i.e. Reactome (Figure 4). Similarly, “counts of kinases implicated in various cancer types” resulted in the most number of hits for *BRAF* (30 cancer types) (Figure 5), which was cross-validated from the COSMIC database.

### ProKinO Application

The compendium of knowledge represented in ProKinO can be used for a variety of applications such as data mining, text mining and genome annotation. In particular, the representation of diverse protein kinase data in machine-readable form enables complex aggregate queries on ontology data, in ways not possible through existing kinase-specific resources. Below, we describe some of these queries to illustrate how ProKinO data can be used for knowledge discovery and hypothesis generation. The queries, which have been formulated in SPARQL, also provide an initial evaluation of ProKinO's usefulness.

#### Query 1

**Figure 6:**
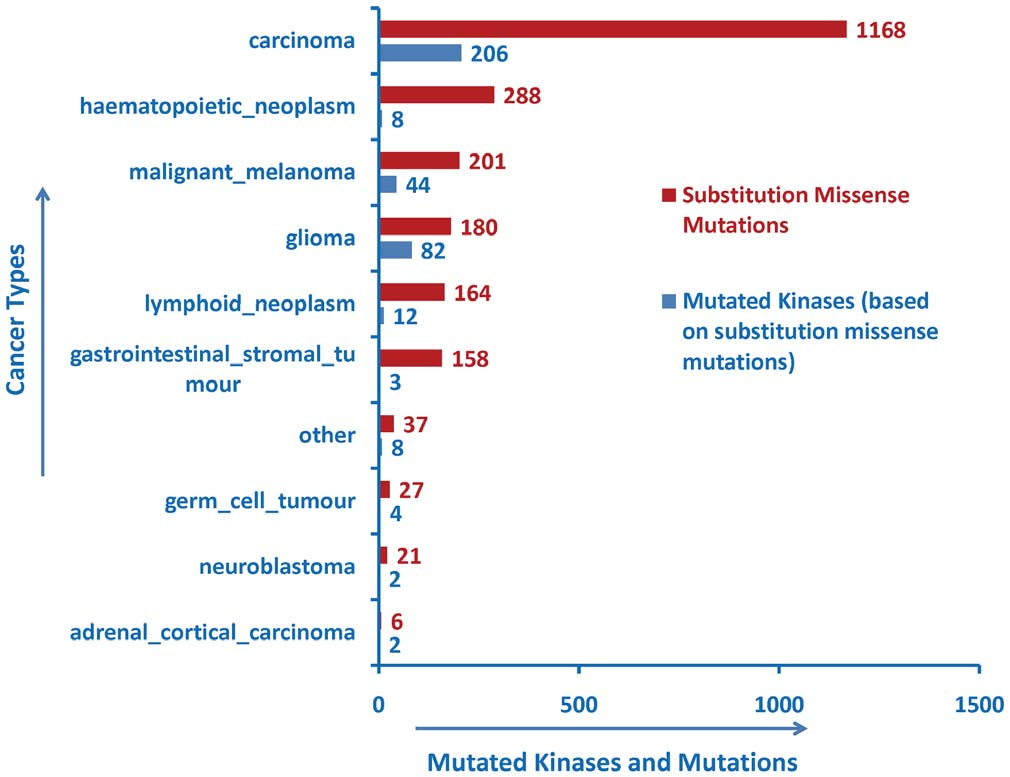
Counts of substitution missense mutations (at least 4) implicated in different types of cancer, and counts of protein kinases having missense mutations implicated in different cancer types. As mentioned in the text, *haematopoietic_neoplasm* has 288 mutations in 8 kinases, while *glioma* has 180 mutations spread over 82 kinases. The SPARQL query to generate this figure can be directly viewed and excuted from the ProKinO browser by selecting “Query 5a” and “Query 5b” under the “Example queries” tab in the main page.

The SPARQL queries “counts of substitution missense mutations in cancer types”, and “counts of protein kinases having missense mutations” was performed on ProKinO to analyze the distribution of kinase mutations in various cancer types. Analysis of the results generated by this query revealed that the distribution of kinase mutations is strikingly different for different cancer types (Figure 6). In particular, *carcinoma* (1168 mutations), *glioma* (180), *malignant melanoma* (201), *haematopoietic neoplasm* (288), and *lymphoid neoplasm* (164) are highly over-represented in kinase mutations compared to other cancer types (Figure 6). Furthermore, the 288 and 164 mutations associated with *haematopoietic neoplasm* and *lymphoid neoplasm* map to only 8 and 12 kinases, respectively. This is in contrast to *glioma*, where the mutations are spread over 82 distinct kinases. While this finding could result from the bias in the sequencing of cancer kinomes from selected cancer types, it is also possible that only a few signaling pathways (associated with the 8 kinases) are altered in *haematopoietic neoplasm*, compared to *glioma*. Such observations have implications in targeting the mutated kinome for therapies, and in generating new hypotheses for experimental studies.

#### Query 2

**Figure 7:**
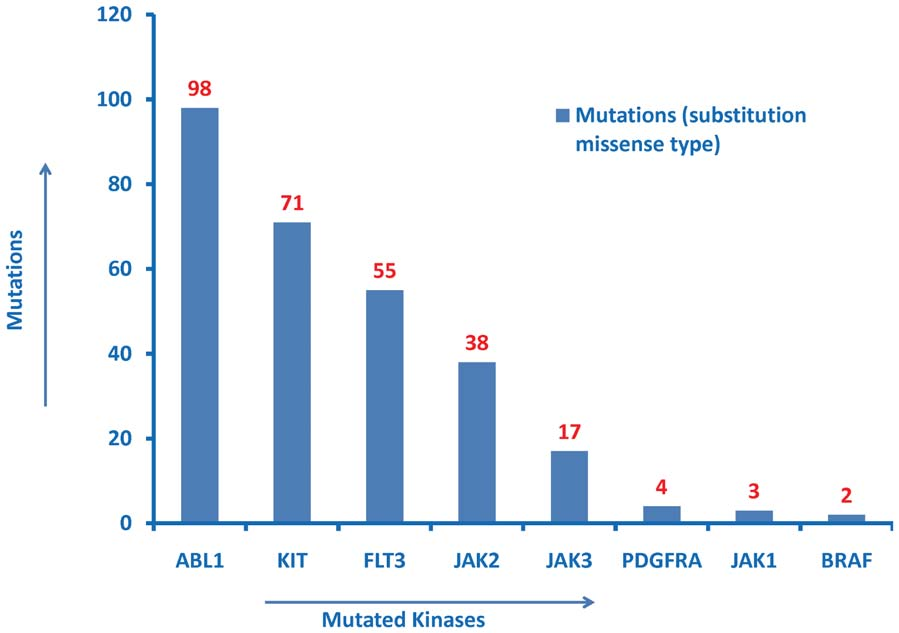
Counts of protein kinases having missense mutations implicated in haematopoietic neoplasm. Top 10 hits in descending order of the counts are displayed. The SPARQL query to generate this figure can be viewed and excuted from the ProKinO browser by selecting “Query 6” under the “Example queries” tab in the main page.

Based on the observation from Query 1, additional SPARQL queries can be performed to obtain further information on the 8 kinases associated with *haematopoietic neoplasm*. For example, the query requesting for the “counts of protein kinases having missense mutations in *haematopoietic neoplasm*” indicates that *ABL1*, *KIT*, *FLT3* and *JAK2* are more frequently mutated compared to other kinases (Figure 7). This observation is consistent with the findings reported in the literature [31], [32], further cross-validating the contents of the ontology.

#### Query 3

Query 2 (above) can be further refined to obtain testable hypotheses regarding cancer mutations. For example, queries requesting functional features and sub-domain location for *ABL1* associated mutations in *haematopoietic neoplasm* revealed that *Y253F* is located in the functionally important *Glycine rich loop* (Sub-domain I; Table S5), and has modified residues property “*Phosphotyrosine*”. With this information, one can formulate a testable hypothesis that “*Y253F* mutation contributes to abnormal *ABL1* functions by altering the phosphorylation status of the glycine rich loop”.

In addition to the queries described above, we have formulated several additional queries on ProKinO. The results obtained from these queries are provided as supplementary figures (see Figures S2, S3, S4, S5, S6, S7, S8, S9). The SPARQL queries themselves are provided in Figure S10.

### Future Directions

ProKinO is an ontology of terms and relationships capturing the state of knowledge on the protein kinase family. Representation of protein kinase knowledge in the form of ontology allows effective mining and systems-level analysis of protein kinase data, as demonstrated through several SPARQL queries. To enable navigation and integrative analysis of ontology data, an ontology browser has been developed. The browser can be accessed from http://vulcan.cs.uga.edu/prokino.

While the current version of ProKinO largely focuses on human protein kinase genes, information on other model organisms can be incorporated in ProKinO through the addition of new classes and data properties in the ontology schema. Likewise, the wealth of information generated on protein kinase substrates through high-throughput phospho-proteomic data can be incorporated to integrate cancer data with proteomics data. Furthermore, we anticipate ProKinO to be useful in providing consistent annotation of mutations identified in cancer genome sequencing studies.

Using specific queries we have demonstrated how data in the ontology can be used to generate new hypotheses regarding the structural and functional impact of mutations. In particular, the observation that nearly 288 mutations map to only eight kinases in *haematopoietic neoplasm* is novel and provides new hypotheses for follow-up studies. Likewise, the prediction that *Y253F* mutation alters the phosphorylation status of the glycine rich loop in ABL tyrosine kinase can be tested experimentally. The SPARQL queries described in this study can be executed from the browser by selecting the “Example queries” tab in the main page.

In the near future, we are planning to submit ProKinO to be included in The Open Biological and Biomedical Ontologies (OBO) foundry (http://www.obofoundry.org/), after introducing necessary changes to make ProKinO conformant to OBO guidelines. Similarly, we plan to make it available through NCBO BioPortal (http://bioportal.bioontology.org/). We also intend to continue the study of the applicability of ProKinO to other bioinformatics tasks, including text mining of bio-medical scientific literature, genome annotation, and others.

## Supporting Information

Figure S1 **Conceptual schema of the Protein Kinase Ontology (ProKinO) showing concepts and relationships representing protein kinase knowledge.** The high resoulution image of the schema is also available from the main page of ProKinO browser.
(PDF)

Figure S2 **Plot showing counts of different mutations (of all types) for all kinase genes.** **Top** 10 hits are displayed in the descending order of their values. Notably, KIT and EGFR are the two of the most frequently mutated kinases in human cancers. The SPARQL query to generate this figure can be directly viewed and excuted from the ProKinO browser by selecting “Query 7” under the “Example query” tab in the main page.
(PDF)

Figure S3 **Plot showing counts of substitution missense mutations for all genes.** Top 10 hits are displayed in descending order of their values. It should be noted that while the total number of mutations is higher for KIT (Figure 2), counting only the missense mutations reveals higher number of mutations for EGFR compared to KIT. The SPARQL query to generate this figure can be directly viewed and excuted from the ProKinO browser by selecting “Query 8” under the “Example query” tab in the main page.
(PDF)

Figure S4 **Plot showing counts of protein kinases (at least 2) having mutations (of any type) implicated in different types of cancer.** Kinases are displayed in descending order of the counts. The SPARQL query to generate this figure can be directly viewed and excuted from the ProKinO browser by selecting “Query 9” under the “Example query” tab in the main page.
(PDF)

Figure S5 **Plot showing counts of protein kinases (at least 4) participating in pathways.** Hits are display in descending order of their values; include only pathways with 4 or more participating kinases. Notably, most of the mutated kinases appear to target pathways associated with the immune system, as indicated by high counts for “signaling in immune system” pathway. The SPARQL query to generate this figure can be directly viewed and excuted from the ProKinO browser by selecting “Query 10” under the “Example query” tab in the main page.
(PDF)

Figure S6 **Plot showing counts of pathways in which mutated protein kinases participate.** Hits are displayed in descending order of their values. Kinases that participate in 4 or more pathways are included. The SPARQL query to generate this figure can be directly viewed and excuted from the ProKinO browser by selecting “Query 11” under the “Example query” tab in the main page.
(PDF)

Figure S7 **Plot showing counts of protein kinases having mutations (of any type) in various primary sites.** Hits are displayed in descending order of their values. Notably, most number of mutated kinases are implicated in the cancers of lung and the central nervous system. The SPARQL query to generate this figure can be directly viewed and excuted from the ProKinO browser by selecting “Query 12” under the “Example query” tab in the main page.
(PDF)

Figure S8 **Plot showing counts of different mutations (all types) for all sub-domains.** Notably, the flanking N and C-terminal tail segment harbor significant number of mutations, followed by the regulatory activation segment and C-helix in the kinase domain. The SPARQL query to generate this figure can be directly viewed and excuted from the ProKinO browser by selecting “Query 13” under the “Example query” tab in the main page.
(PDF)

Figure S9 **Plot showing counts of substitution missense mutations of the protein kinase FLT3 all having the primary site of** ***Haematopoietic*** **and** ***Lymphoid tissue*** **, and located in various sub-domains.** Notably, the activation segment has the most number of mutations. The SPARQL query to generate this figure can be directly viewed and excuted from the ProKinO browser by selecting “Query 14” under the “Example query” tab in the main page.
(PDF)

Figure S10 **SPARQL queries for the main (** **Figures 2** **,** **3** **,** **4** **,** **5** **,** **6** **,** **7** **) and supplementary (figures S2, S3, S4, S5, S6, S7, S8, S9) figures described in this study.**
(PDF)

Table S1 **Classes (concepts) in ProKinO.**
(DOC)

Table S2 **Object properties used in ProKinO.**
(DOC)

Table S3 **Data properties used in ProKinO.**
(DOC)

Table S4 **ProKinO evaluation statistics.**
(DOC)

Table S5 **Protein kinases implicated in** ***Haematopoietic and lymphoid_tissue*** **and having modified residue type property.**
(DOC)
