# Supplementary figures and images for: ProKinO: An Ontology for Integrative Analysis of Protein Kinases in Cancer
Source: PLoS One. 2011 Dec 14;6(12):e28782. doi: 10.1371/journal.pone.0028782 (PMC3237543; doi:10.1371/journal.pone.0028782)

## Figure S1

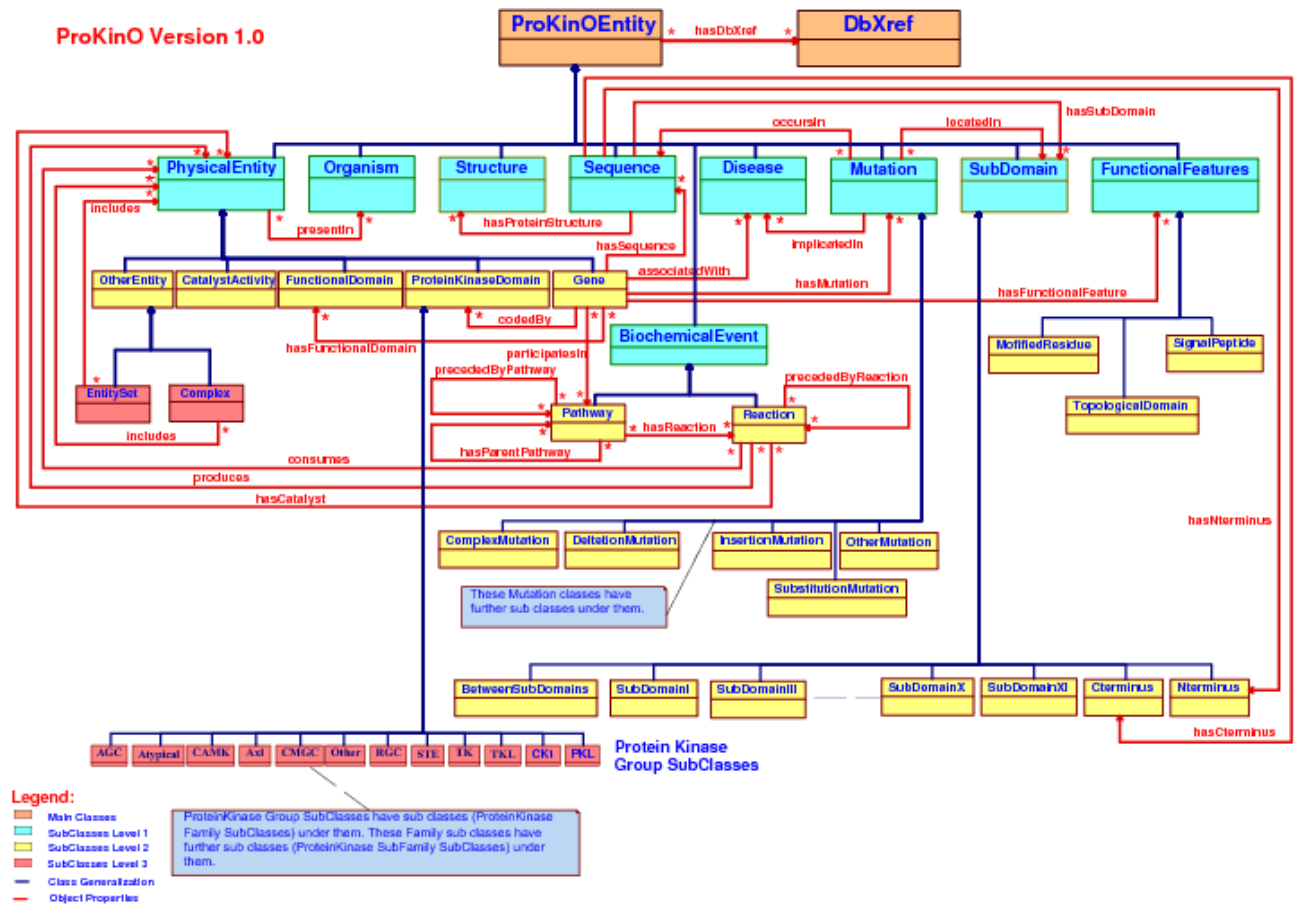

Supplement: Figure S1 — Conceptual schema of the Protein Kinase Ontology (ProKinO) showing concepts and relationships representing protein kinase knowledge. The high resoulution image of the schema is also available from the main page of ProKinO browser. (PDF) [file pone.0028782.s001.pdf]

Figure S2

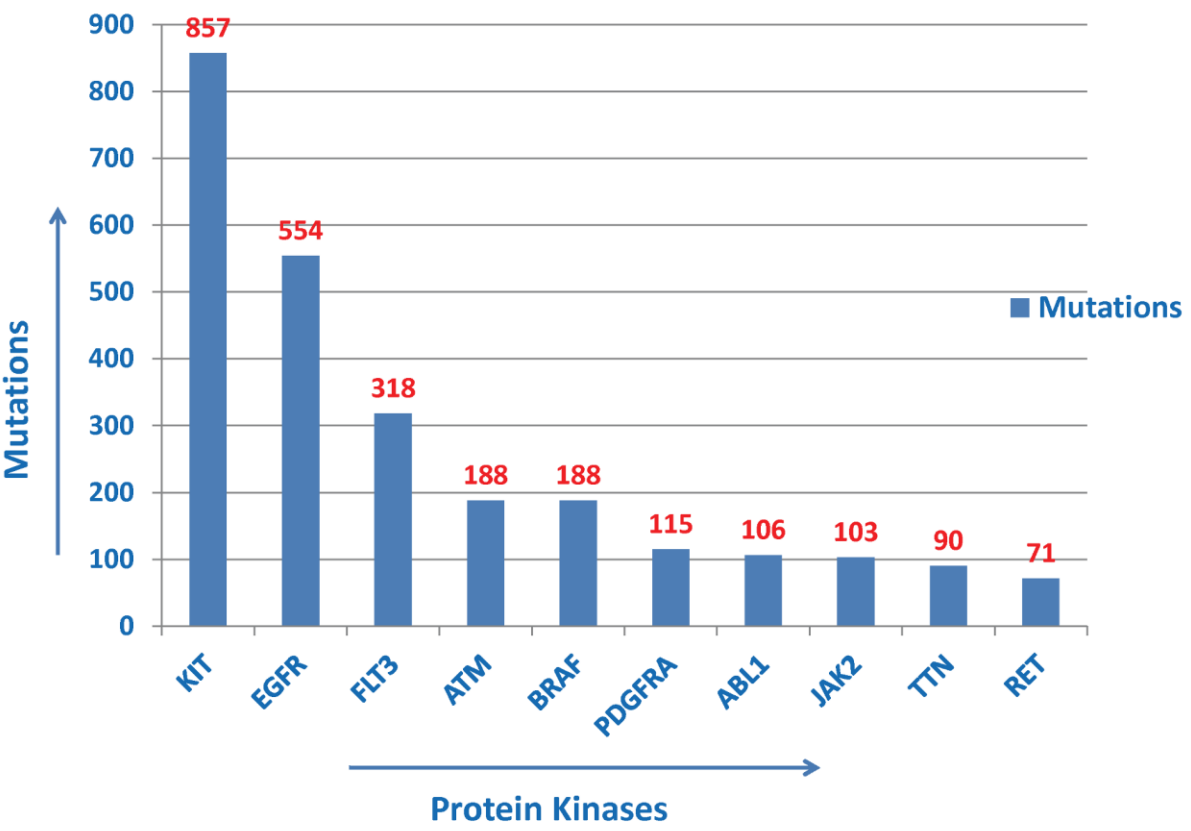

Supplement: Figure S2 — Plot showing counts of different mutations (of all types) for all kinase genes. Top 10 hits are displayed in the descending order of their values. Notably, KIT and EGFR are the two of the most frequently mutated kinases in human cancers. The SPARQL query to generate this figure can be directly viewed and excuted from the ProKinO browser by selecting “Query 7” under the “Example query” tab in the main page. (PDF) [file pone.0028782.s002.pdf]

**Figure S3**

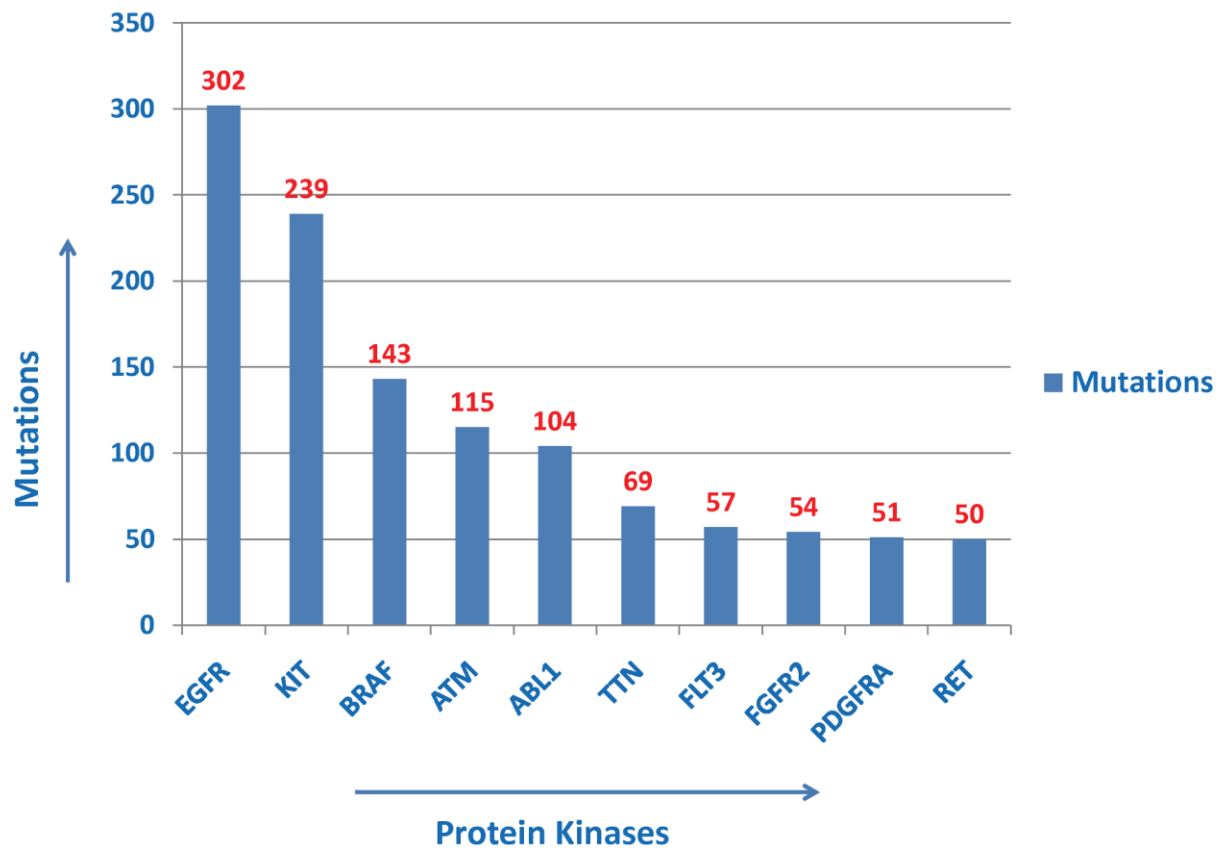

Supplement: Figure S3 — Plot showing counts of substitution missense mutations for all genes. Top 10 hits are displayed in descending order of their values. It should be noted that while the total number of mutations is higher for KIT (Figure 2), counting only the missense mutations reveals higher number of mutations for EGFR compared to KIT. The SPARQL query to generate this figure can be directly viewed and excuted from the ProKinO browser by selecting “Query 8” under the “Example query” tab in the main page. (PDF) [file pone.0028782.s003.pdf]

Figure S4

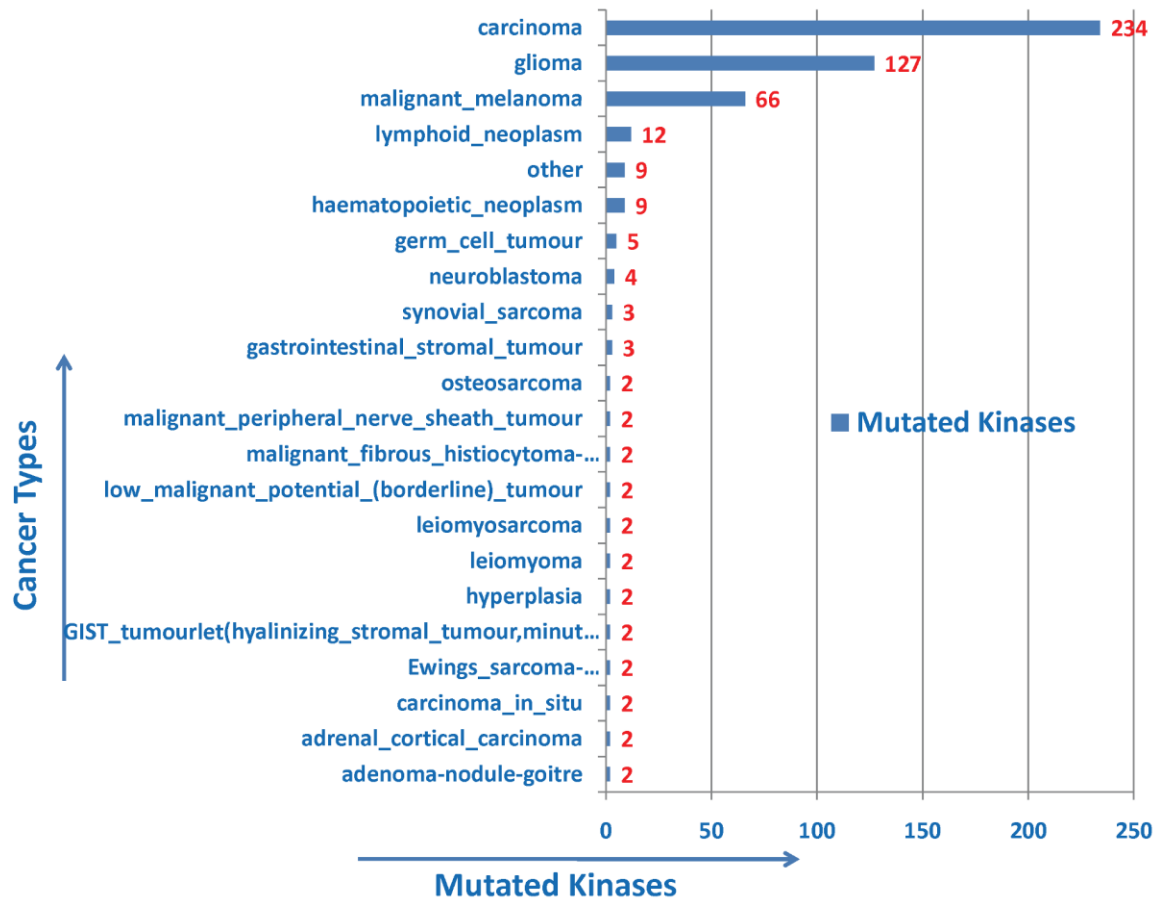

Supplement: Figure S4 — Plot showing counts of protein kinases (at least 2) having mutations (of any type) implicated in different types of cancer. Kinases are displayed in descending order of the counts. The SPARQL query to generate this figure can be directly viewed and excuted from the ProKinO browser by selecting “Query 9” under the “Example query” tab in the main page. (PDF) [file pone.0028782.s004.pdf]

Figure S5

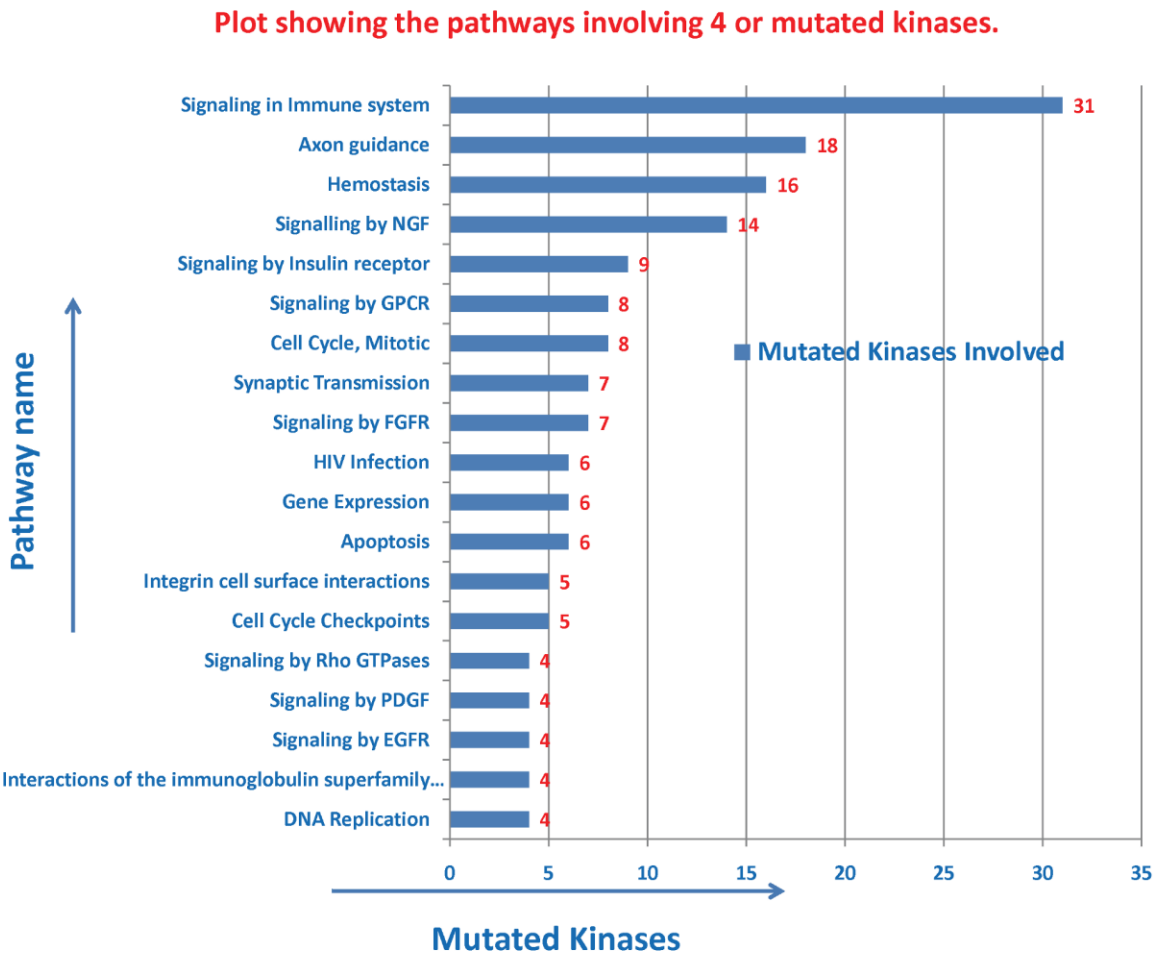

Supplement: Figure S5 — Plot showing counts of protein kinases (at least 4) participating in pathways. Hits are display in descending order of their values; include only pathways with 4 or more participating kinases. Notably, most of the mutated kinases appear to target pathways associated with the immune system, as indicated by high counts for “signaling in immune system” pathway. The SPARQL query to generate this figure can be directly viewed and excuted from the ProKinO browser by selecting “Query 10” under the “Example query” tab in the main page. (PDF) [file pone.0028782.s005.pdf]

Figure S6

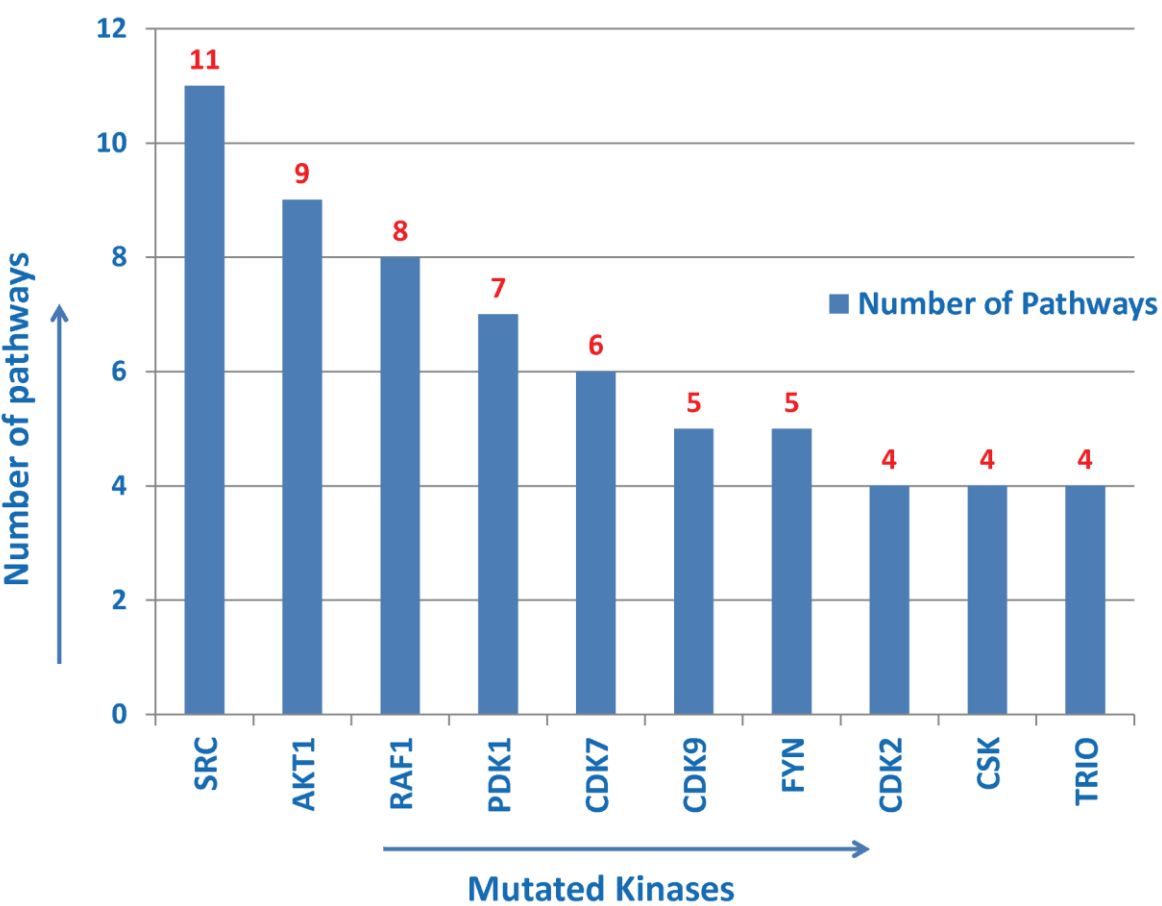

Supplement: Figure S6 — Plot showing counts of pathways in which mutated protein kinases participate. Hits are displayed in descending order of their values. Kinases that participate in 4 or more pathways are included. The SPARQL query to generate this figure can be directly viewed and excuted from the ProKinO browser by selecting “Query 11” under the “Example query” tab in the main page. (PDF) [file pone.0028782.s006.pdf]

**Figure S7**

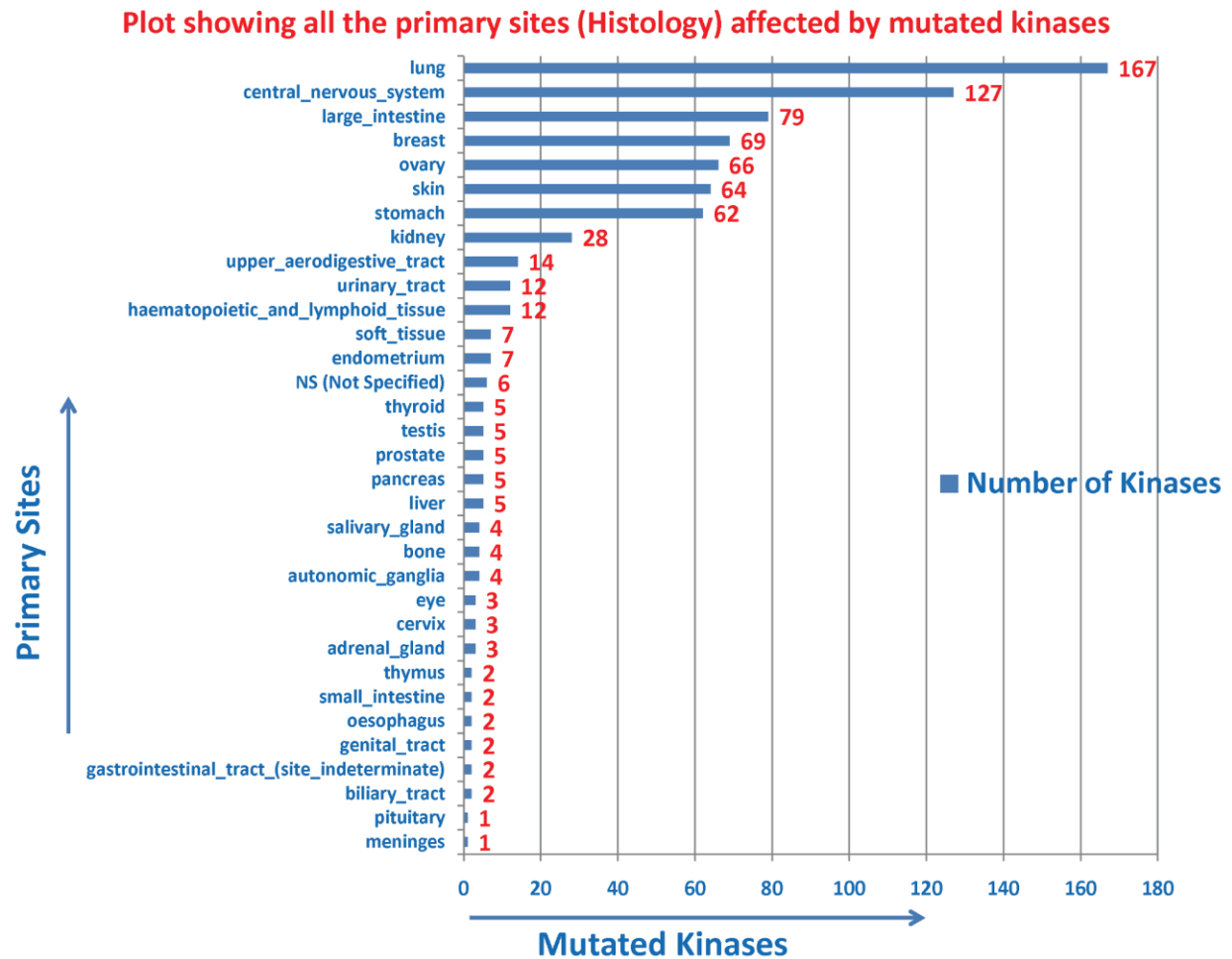

Supplement: Figure S7 — Plot showing counts of protein kinases having mutations (of any type) in various primary sites. Hits are displayed in descending order of their values. Notably, most number of mutated kinases are implicated in the cancers of lung and the central nervous system. The SPARQL query to generate this figure can be directly viewed and excuted from the ProKinO browser by selecting “Query 12” under the “Example query” tab in the main page. (PDF) [file pone.0028782.s007.pdf]

**Figure S8**

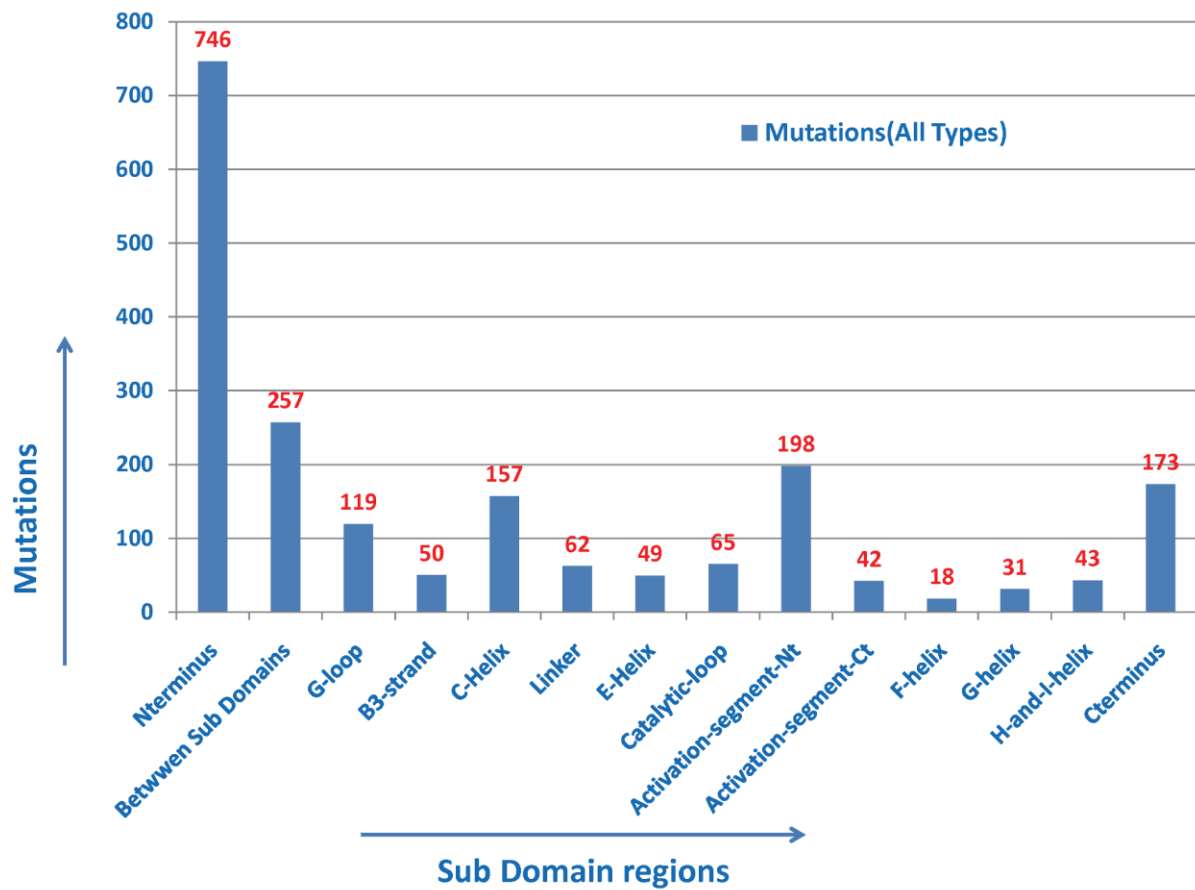

Supplement: Figure S8 — Plot showing counts of different mutations (all types) for all sub-domains. Notably, the flanking N and C-terminal tail segment harbor significant number of mutations, followed by the regulatory activation segment and C-helix in the kinase domain. The SPARQL query to generate this figure can be directly viewed and excuted from the ProKinO browser by selecting “Query 13” under the “Example query” tab in the main page. (PDF) [file pone.0028782.s008.pdf]

Figure S9

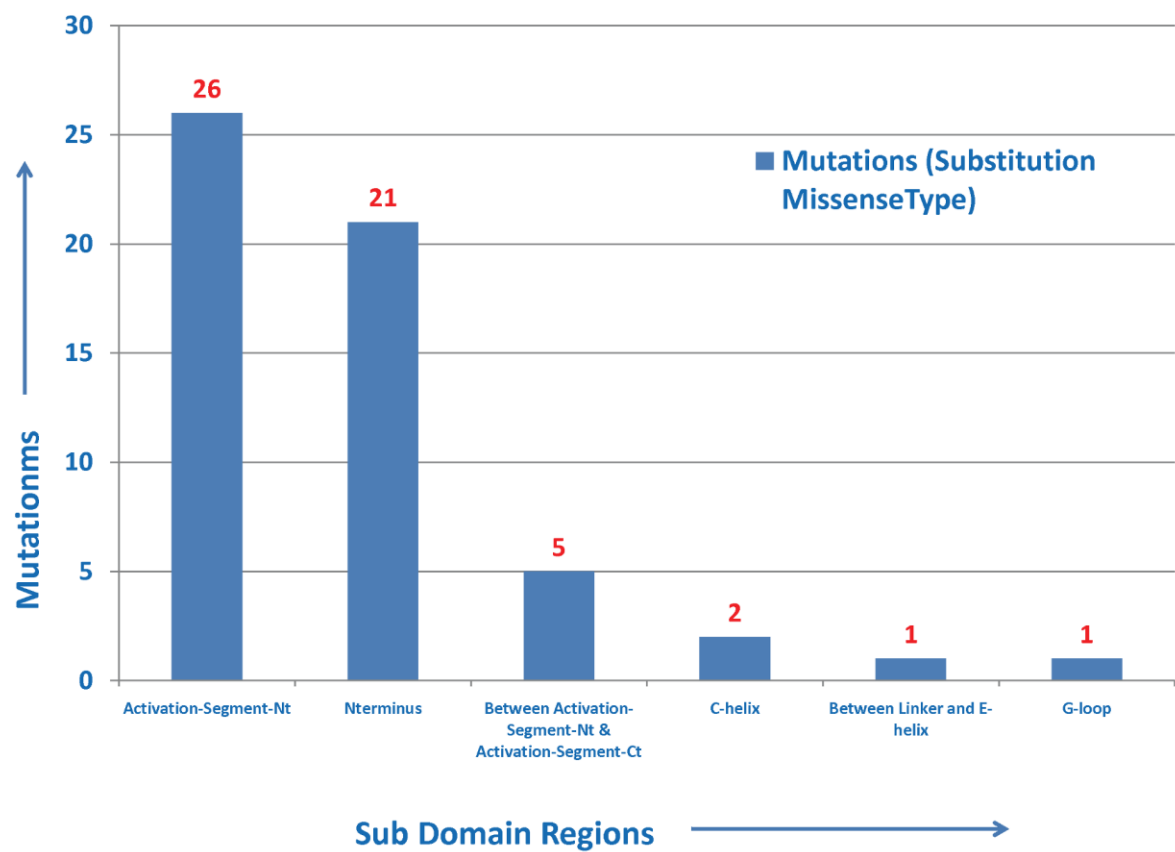

Supplement: Figure S9 — Plot showing counts of substitution missense mutations of the protein kinase FLT3 all having the primary site of Haematopoietic and Lymphoid tissue , and located in various sub-domains. Notably, the activation segment has the most number of mutations. The SPARQL query to generate this figure can be directly viewed and excuted from the ProKinO browser by selecting “Query 14” under the “Example query” tab in the main page. (PDF) [file pone.0028782.s009.pdf]
