# Supplementary material for: ProKinO: An Ontology for Integrative Analysis of Protein Kinases in Cancer
Source: PLoS One. 2011 Dec 14;6(12):e28782. doi: 10.1371/journal.pone.0028782 (PMC3237543; doi:10.1371/journal.pone.0028782)
Supplement: Figure S10 — SPARQL queries for the main ( Figures 2 , 3 , 4 , 5 , 6 , 7 ) and supplementary (figures S2, S3, S4, S5, S6, S7, S8, S9) figures described in this study. (PDF) [file pone.0028782.s010.pdf]

Figure S10

Query 1 (Figure 2): Find the counts of crystal structures for all protein kinases and display top 10 of them in descending order of their counts.

```
PREFIX    rdf: <http://www.w3.org/1999/02/22-rdf-syntax-ns#>
PREFIX   rdfs: <http://www.w3.org/2000/01/rdf-schema#>
PREFIX prokino: <http://om.cs.uga.edu/prokino/1.0/#>
SELECT (STR(?geneName) As ?Gene_Name) (COUNT(*) AS ?No_Structures) WHERE
{
  ?gene  rdf:type                  prokino:Gene .
  ?gene  prokino:hasPrimaryName      ?geneName .
  ?gene  prokino:hasSequence        ?seq .
  ?seq   prokino:hasProteinStructure ?struct
}
GROUP BY ?geneName
ORDER BY DESC(?No_Structures)
LIMIT 10
```

Query 2 (Figure 3): Find the counts of isoforms for all protein kinases and display top 10 of them in descending order of their values

```
PREFIX    rdf: <http://www.w3.org/1999/02/22-rdf-syntax-ns#>
PREFIX   rdfs: <http://www.w3.org/2000/01/rdf-schema#>
PREFIX prokino: <http://om.cs.uga.edu/prokino/1.0/#>
SELECT (STR(?geneName) AS ?Gene_Name) (COUNT(*) AS ?No_Sequences) WHERE
{
  ?gene  rdf:type              prokino:Gene .
  ?gene  prokino:hasPrimaryName ?geneName .
  ?gene  prokino:hasSequence    ?seq .
  filter( regex( str(?seq), "Isoform" ) || regex( str(?seq), "UniProt" ) )
}
GROUP BY ?geneName
ORDER BY DESC(?No_Sequences)
LIMIT 10
```

Query 3 (Figure 4) Counts of number of pathways associated with all protein kinases. Top 10 kinases with the most number of pathways are displayed in descending order.

```
PREFIX    rdf: <http://www.w3.org/1999/02/22-rdf-syntax-ns#>
PREFIX   rdfs: <http://www.w3.org/2000/01/rdf-schema#>
PREFIX prokino: <http://om.cs.uga.edu/prokino/1.0/#>
SELECT (STR(?geneName) AS ?Gene_Name) (COUNT(*) AS ?No_Pathways) WHERE
```

```
{
  ?gene rdf:type              prokino:Gene .
  ?gene prokino:hasPrimaryName ?geneName .
  {
    SELECT ?gene ?pathway WHERE
    {
       ?gene    rdf:type              prokino:Gene .
       ?gene    prokino:participatesIn ?pathway .
    }
    GROUP BY ?gene ?pathway
  }
}
GROUP BY ?geneName
ORDER BY DESC(?No_Pathways)
LIMIT 10
```

Query 4 (Figure 5) Find the counts of different cancer types implicated in protein kinases and display top 10 of them in descending order of their values.

```
PREFIX     rdf: <http://www.w3.org/1999/02/22-rdf-syntax-ns#>
PREFIX    rdfs: <http://www.w3.org/2000/01/rdf-schema#>
PREFIX prokino: <http://om.cs.uga.edu/prokino/1.0/#>
SELECT (STR(?geneName) AS ?Gene_Name) (COUNT(*) AS ?No_CancerTypes) WHERE
{
  ?gene rdf:type              prokino:Gene .
  ?gene prokino:hasPrimaryName ?geneName .
  {
    SELECT ?gene ?cancer WHERE
    {
       ?gene  rdf:type            prokino:Gene .
       ?gene  prokino:hasMutation  ?mut .
       ?mut   prokino:implicatedIn ?cancer
    }
    GROUP BY ?gene ?cancer
  }
}
GROUP BY ?geneName
ORDER BY DESC(?No_CancerTypes)
LIMIT 10
```

Query 5a (Figure 6): Find the counts of substitution missense mutations (at least 4) implicated in different types of cancer, and display them in descending order of the counts.

```
PREFIX     rdf: <http://www.w3.org/1999/02/22-rdf-syntax-ns#>
PREFIX    rdfs: <http://www.w3.org/2000/01/rdf-schema#>
PREFIX prokino: <http://om.cs.uga.edu/prokino/1.0/#>
```

```
SELECT (STR(?cancerName) AS ?CancerType) (COUNT(*) AS ?No_Mutations)
WHERE
{
  ?gene   rdf:type                     prokino:Gene .
  ?gene   prokino:hasMutation           ?mut .
  ?mut    prokino:implicatedIn          ?cancer .
  ?mut    rdf:type                     prokino:Missense .
  ?cancer rdfs:label                    ?cancerName
}
GROUP BY ?cancerName
HAVING (count(distinct *) >= 4)
ORDER BY DESC(?No_Mutations)
```

Query 5b (Figure 6): Find the counts of protein kinases having missense mutations implicated in different types of cancer, and display them in descending order of the counts.

```
PREFIX     rdf: <http://www.w3.org/1999/02/22-rdf-syntax-ns#>
PREFIX    rdfs: <http://www.w3.org/2000/01/rdf-schema#>
PREFIX prokino: <http://om.cs.uga.edu/prokino/1.0/#>
SELECT (str(?cancerName) AS ?CancerType) (COUNT(*) AS ?No_Genes) WHERE
{
 ?cancer rdf:type   prokino:Cancer .
 ?cancer rdfs:label ?cancerName

 {
   SELECT ?gene ?cancer WHERE
   {
      ?gene   rdf:type                     prokino:Gene .
      ?gene   prokino:hasMutation           ?mut .
      ?mut    prokino:implicatedIn          ?cancer .
      ?mut    rdf:type                     prokino:Missense .
   }
   GROUP BY ?gene ?cancer
 }
}
GROUP BY ?cancer ?cancerName
HAVING (count(distinct *) >= 2)
ORDER BY desc(?No_Genes)
```

Query 6 (Figure 7): Find the counts of protein kinases having missense mutations implicated in haematopoietic neoplasm and display top 10 of them in descending order of the counts

```
PREFIX     rdf: <http://www.w3.org/1999/02/22-rdf-syntax-ns#>
PREFIX    rdfs: <http://www.w3.org/2000/01/rdf-schema#>
PREFIX prokino: <http://om.cs.uga.edu/prokino/1.0/#>


SELECT (STR(?geneName) AS ?Gene_Name) (COUNT(*) AS ?No_Mutations) WHERE
{
  ?gene  rdf:type                prokino:Gene .
  ?gene  prokino:hasPrimaryName  ?geneName .
  ?gene  prokino:hasMutation     ?mut .
  ?mut   prokino:implicatedIn    prokino:haematopoietic_neoplasm .
  ?mut   rdf:type                prokino:Missense .
}
GROUP BY ?geneName
ORDER BY DESC(?No_Mutations)
LIMIT 10
```

Query 7 (Suppl. Figure 2): Find the counts of different mutations (of all types) for all kinase genes and display the top 10 in the descending order

```
PREFIX     rdf: <http://www.w3.org/1999/02/22-rdf-syntax-ns#>
PREFIX    rdfs: <http://www.w3.org/2000/01/rdf-schema#>
PREFIX prokino: <http://om.cs.uga.edu/prokino/1.0/#>
SELECT (STR(?geneName) AS ?Gene_Name) (COUNT(*) AS ?No_Mutations) WHERE
{
    ?gene  rdf:type               prokino:Gene .
    ?gene  prokino:hasMutation    ?mut .
    ?gene  prokino:hasPrimaryName ?geneName .
}
GROUP BY ?gene ?geneName
ORDER BY DESC(?No_Mutations)
LIMIT 10
```

Query 8 (Suppl. Figure 3): Find the counts of substitution missense mutations for all genes and display top 10 of them in descending order

```
PREFIX     rdf: <http://www.w3.org/1999/02/22-rdf-syntax-ns#>
PREFIX    rdfs: <http://www.w3.org/2000/01/rdf-schema#>
PREFIX prokino: <http://om.cs.uga.edu/prokino/1.0/#>
SELECT (STR(?geneName) AS ?Gene_Name) (COUNT(*) AS ?No_Mutations) WHERE
{
  ?gene  rdf:type                prokino:Gene .
  ?gene  prokino:hasMutation     ?mut .
  ?gene  prokino:hasPrimaryName  ?geneName .
  ?mut   rdf:type                prokino:Missense .
}
```

```
GROUP BY ?gene ?geneName
ORDER BY DESC(?No_Mutations)
LIMIT 10
```

Query 9 (Suppl. Figure 4): Find the counts of protein kinases (at least 2) having mutations (of any type) implicated in different types of cancer and display them in descending order

```
PREFIX    rdf: <http://www.w3.org/1999/02/22-rdf-syntax-ns#>
PREFIX   rdfs: <http://www.w3.org/2000/01/rdf-schema#>
PREFIX prokino: <http://om.cs.uga.edu/prokino/1.0/#>
SELECT (str(?cancername) AS ?Cancer_Type) (COUNT(*) AS ?No_Genes) WHERE
{
  ?cancer rdf:type   prokino:Cancer .
  ?cancer rdfs:label ?cancername .
  {
    SELECT ?gene ?cancer WHERE
    {
      ?gene  rdf:type                prokino:Gene .
      ?gene  prokino:hasMutation       ?mut .
      ?mut   prokino:implicatedIn      ?cancer .
    }
    GROUP BY ?gene ?cancer
  }
}
GROUP BY ?cancer ?cancername
HAVING (count(distinct *) >= 2)
ORDER BY desc(?No_Genes)
```

Query 10 (Suppl. Figure 5): Find the counts of protein kinases (at least 4) participating in pathways and display them in descending order; include only pathways with 4 or more participating kinases

```
PREFIX    rdf: <http://www.w3.org/1999/02/22-rdf-syntax-ns#>
PREFIX   rdfs: <http://www.w3.org/2000/01/rdf-schema#>
PREFIX prokino: <http://om.cs.uga.edu/prokino/1.0/#>
SELECT (STR(?pathwayname) AS ?Pathway) (COUNT(*) AS ?No_Genes) WHERE
{
  ?pathway rdf:type            prokino:Pathway .
  ?pathway prokino:hasPrimaryName ?pathwayname .
  {
    SELECT ?pathway ?mut ?gene WHERE
    {
      ?gene   rdf:type            prokino:Gene .
      ?gene   prokino:hasMutation   ?mut .
      ?gene   prokino:participatesIn ?pathway .
    }
    GROUP BY ?pathway ?gene
  }
}
```

```
GROUP BY ?pathway ?pathwayname
HAVING (count(distinct *) >= 4)
ORDER BY DESC(?No_Genes)
```

Query 11 (Suppl. Figure 6). Find the counts of pathways in which mutated protein kinases pariticipate and display them in descending order; include only kinases with 4 or more pathways in which they participate

```
PREFIX    rdf: <http://www.w3.org/1999/02/22-rdf-syntax-ns#>
PREFIX   rdfs: <http://www.w3.org/2000/01/rdf-schema#>
PREFIX prokino: <http://om.cs.uga.edu/prokino/1.0/#>
SELECT (STR(?geneName) AS ?Gene_Name) (COUNT(*) AS ?No_Pathways) WHERE
{
  ?gene rdf:type              prokino:Gene .
  ?gene prokino:hasPrimaryName ?geneName .
  {
    SELECT ?gene ?pathway WHERE
    {
      ?gene   rdf:type              prokino:Gene .
      ?gene   prokino:hasMutation    ?mut .
      ?gene   prokino:participatesIn ?pathway .
    }
    GROUP BY ?gene ?pathway
  }
}
GROUP BY ?geneName
HAVING (count(distinct *) >= 4)
ORDER BY DESC(?No_Pathways)
```

Query 12 (Suppl. Figure 7): Find the counts of protein kinases having mutations (of any type) in various primary sites and display them in descending order

```
PREFIX    rdf: <http://www.w3.org/1999/02/22-rdf-syntax-ns#>
PREFIX   rdfs: <http://www.w3.org/2000/01/rdf-schema#>
PREFIX prokino: <http://om.cs.uga.edu/prokino/1.0/#>
SELECT (STR(?primarysite) AS ?PrimarySite) (COUNT(*) AS ?No_Genes) WHERE
{
  ?gene  rdf:type  prokino:Gene .
  {
    SELECT ?gene ?primarysite WHERE
    {
      ?gene  rdf:type              prokino:Gene .
      ?gene  prokino:hasMutation    ?mut .
      ?mut   prokino:hasPrimarySite ?primarysite .
    }
    GROUP BY ?primarysite ?gene
  }
}
```

```
GROUP BY ?primarysite
ORDER BY DESC(?No_Genes)
```

Query 13 (Suppl. Figure 8): Find the counts of different mutations (all types) for all sub-domains and display them in descending order

```
PREFIX    rdf: <http://www.w3.org/1999/02/22-rdf-syntax-ns#>
PREFIX   rdfs: <http://www.w3.org/2000/01/rdf-schema#>
PREFIX prokino: <http://om.cs.uga.edu/prokino/1.0/#>
SELECT (STR(?subdomainName) AS ?Subdomain_Name) (COUNT(*) AS ?No_Mutations) WHERE
{
   ?gene      rdf:type           prokino:Gene .
   ?gene      prokino:hasMutation ?mutation .
   ?mutation  prokino:locatedIn   ?subdomain .
   ?subdomain rdf:type           ?subdomainType .
   ?subdomainType rdfs:label      ?subdomainName
}
GROUP BY ?subdomainType ?subdomainName
ORDER BY DESC(?No_Mutations)
```

Query 14 (Suppl. Figure 9): Find the counts of substitution missense mutations of the protein kinase FLT3 all having the primary site of Haematopoietic and Lymphoid tissue, and located in various sub-domains and display them in descending order.

```
PREFIX    rdf: <http://www.w3.org/1999/02/22-rdf-syntax-ns#>
PREFIX   rdfs: <http://www.w3.org/2000/01/rdf-schema#>
PREFIX prokino: <http://om.cs.uga.edu/prokino/1.0/#>
PREFIX    afn: <http://jena.hpl.hp.com/ARQ/function#>
SELECT (STR(?subdomainName) AS ?Subdomain_Name)
     ?Between_Subdomains_Details
     (COUNT(*) AS ?No_Mutations) WHERE
{
  prokino:FLT3   prokino:hasMutation     ?mutation .
  ?mutation      prokino:locatedIn       ?subdomain .
  ?mutation      prokino:hasPrimarySite  "haematopoietic_and_lymphoid_tissue" .
  ?mutation      rdf:type                prokino:Missense .
  ?subdomain     rdf:type                ?subdomainType .
  ?subdomainType rdfs:label              ?subdomainName
  OPTIONAL {
    ?subdomain rdf:type    prokino:BetweenSubDomains .
    ?subdomain rdfs:label  ?betweenType
    BIND( afn:substring(STR(?betweenType), 22) AS ?Between_Subdomains_Details )
  }
}
GROUP BY ?subdomainType ?subdomainName ?Between_Subdomains_Details
ORDER BY DESC(?No_Mutations)
```
