# Supplementary material for: ProKinO: An Ontology for Integrative Analysis of Protein Kinases in Cancer
Source: PLoS One. 2011 Dec 14;6(12):e28782. doi: 10.1371/journal.pone.0028782 (PMC3237543; doi:10.1371/journal.pone.0028782)
Supplement: Table S1 — Classes (concepts) in ProKinO. (DOC) [file pone.0028782.s011.doc]

**Table S1.** Classes (concepts) in ProKinO.

| **Class Name** | **Super Class Name** | **Sub Class Name** |
| --- | --- | --- |
| **Parent Level** | | |
| DbXref | N/A | --- |
| ProKinOEntity | N/A | BiochemicalEvent, Disease, FunctionalFeature, Mutation, Organism, PhysicalEntity  Sequence, Structure  SubDomain |
| **Next Level of Heirarchy** | | |
| BiochemicalEvent | ProKinOEntity | Pathway, Reaction |
| Disease | ProKinOEntity | Cancer |
| FunctionalFeature | ProKinOEntity | ModifiedResidue, SignalPeptide, TopologicalDomain |
| Mutation | ProKinOEntity | ComplexMutation, DeletionMutation, InsertionMutation, OtherMutation, SubstitutionMutation |
| Organism | ProKinOEntity | Human |
| PhysicalEntity | ProKinOEntity | FunctionalDomain, Gene, OtherEntity, ProteinKinaseDomain |
| Sequence | ProKinOEntity | --- |
| Structure | ProKinOEntity | --- |
| SubDomain | ProKinOEntity | BetweenSubDomains, Cterminus, Nterminus, SubDomain (I to XI) |
| **Next Level of Heirarchy** | | |
| Pathway | BiochemicalEvent | --- |
| Reaction | BiochemicalEvent | --- |
| Cancer | Disease | --- |
| ModifiedResidue | FunctionalFeature | --- |
| SignalPeptide | FunctionalFeature | --- |
| TopologicalDomain | FunctionalFeature | --- |
| ComplexMutation | Mutation | ComplexDeletionInframe, ComplexFrameshift, ComplexInsertionInframe, CompoundSubstitution, DeletionInframe |
| DeletionMutation | Mutation | DeletionFrameshift, DeletionInframe |
| InsertionMutation | Mutation | InsertionFrameshift  InsertionInframe |
| OtherMutation | Mutation | --- |
| SubstitutionMutation | Mutation | CodingSilent, Missense, Nonsense |
| Human | Organism | --- |
| FunctionalDomain | PhysicalEntity | --- |
| Gene | PhysicalEntity | --- |
| OtherEntity | PhysicalEntity | CatalystActivity, Complex, EntitySet |
| ProteinKinaseDomain | PhysicalEntity | AGCgroup, Atypicalgroup, CAMKgroup, CK1group, CMGCgroup, NDKgroup, Othergroup, PKLgroup, RGCgroup, STEgroup, TKgroup, TKLgroup |
| BetweenSubDomains | SubDomain | --- |
| Cterminus | SubDomain | --- |
| Nterminus | SubDomain | --- |
| SubDomain (I to XI) | SubDomain | --- |
| **Next Level of Heirarchy** | | |
| AGCgroup | ProteinKinaseDomain | [Aktfamily, GRKfamily, DMPKfamily, NDRfamily, MASTfamily, RSKfamily, PDK1family, PKAfamily, PKCfamily, PKGfamily, PKNfamily, RSKLfamily  SGKfamily, RSKRfamily, YANKfamily] * |
| Atypicalgroup | ProteinKinaseDomain | [ABC1family Alphafamily, PIKKfamily, PDHKfamily, BCRfamily, BRDfamily, FASTfamily, G11family, RIOfamily, TAF1family, TIF1family, GTF2F1family  Col4A3BPfamily, BLVRAfamily, BAZfamily]* |
| CAMKgroup | ProteinKinaseDomain | [CAMKLfamily, CAMK1family, CAMK2family, MLCKfamily, CASKfamily, RAD53family, DAPKfamily, MAPKAPKfamily, Triofamily, PHKfamily, PIMfamily, PKDfamily, PSKfamily, SgK495family, TSSKfamily, STK33family, Trblfamily, CAMK-Uniquefamily, DCAMKLfamily]* |
| CK1group | ProteinKinaseDomain | [CK1family, TTBKfamily, VRKfamily]* |
| CMGCgroup | ProteinKinaseDomain | [CDKfamily, CDKLfamily, CK2family, CLKfamily, DYRKfamily, MAPKfamily, GSKfamily, RCKfamily, SRPKfamily]* |
| NDKgroup | ProteinKinaseDomain | NDKfamily |
| Othergroup | ProteinKinaseDomain | [NAKfamily, Aurfamily, BUBfamily, CAMKKfamily, CDC7family, NKF4family, ULKfamily, PEKfamily, Haspinfamily, IKKfamily, IREfamily, KISfamily, MOSfamily, WEEfamily, NEKfamily, NRBPfamily, TOPKfamily, VPS15family, NKF2family, PLKfamily, Bud32family, WNKfamily, Other-Uniquefamily, NKF1family, SCY1family, SgK071family, NKF3family, NKF5family, SgK493family, Dustyfamily, Slobfamily, TBCKfamily, TLKfamily, TTKfamily, PAN3family]* |
| PKLgroup | ProteinKinaseDomain | FJfamily |
| RGCgroup | ProteinKinaseDomain | RGCfamily |
| STEgroup | ProteinKinaseDomain | [STE-Uniquefamily, STE20family, STE7family, STE11family]* |
| TKgroup | ProteinKinaseDomain | [Ablfamily, Ackfamily, ALKfamily, Axlfamily, Srcfamily, Tecfamily, CCK4family, Cskfamily, DDRfamily, EGFRfamily, Ephfamily, FAKfamily, Ferfamily, FGFRfamily, VEGFRfamily, PDGFRfamily, InsRfamily, Jakfamily, Lmrfamily, Metfamily, Muskfamily, Retfamily, Rorfamily, Sevfamily, Rykfamily, TK-Uniquefamily, Sykfamily, Tiefamily, Trkfamily]* |
| TKLgroup | ProteinKinaseDomain | [STKRfamily, RIPKfamily, RAFfamily, MLKfamily, IRAKfamily, LISKfamily, LRRKfamily, TKL-Uniquefamily]* |

*****These family classes further have *(subfamilyname) subfamily* as sub classes under them.
