# Supplementary material for: ProKinO: An Ontology for Integrative Analysis of Protein Kinases in Cancer
Source: PLoS One. 2011 Dec 14;6(12):e28782. doi: 10.1371/journal.pone.0028782 (PMC3237543; doi:10.1371/journal.pone.0028782)
Supplement: Table S2 — Object properties used in ProKinO. (DOC) [file pone.0028782.s012.doc]

**Table S2.** Object properties used in ProKinO.

| **Relationship**  **(Object Property)** | **Domain Class** | **Range Class** | **Example** |
| --- | --- | --- | --- |
| *associatedWith* | Gene | Disease | EGFR is ***associatedWith*** carcinoma. |
| *codedBy* | ProteinKinaseDomain | Gene | AKT1domain is ***codedBy*** AKT1. |
| *consumes* | Reaction | PhysicalEntity | “EGFR dimerization” ***consumes*** “EGF:EGFR [plasma membrane]”. |
| *hasCatalyst* | Reaction | PhysicalEntity | "Phosphorylation of AKT2 by PDK1" ***hasCatalyst*** "kinase activity of PIP3:PDK1 complex [plasma membrane] [plasma membrane]". |
| *hasCterminus* | Sequence | Cterminus | “Seq-EGFR-UniProt” (EGFR UniProt sequence) ***hasCterminus*** “EGFR-UniProt-Cterminus” (Cterminus having start position 968). |
| *hasDbXref* | ProKinOEntity | hasDbXref | EGFR ***hasDbXref*** *“*UniProt-P00533” (external cross reference to UniProt). |
| *hasFunctionalDomain* | Gene | hasFunctionalDomain | EGFR ***hasFunctionalDomain*** “Recep_L_domain”. |
| *hasFunctionalFeature* | Gene | hasFunctionalFeature | EGFR ***hasFunctionalFeature****“*EGFR- tmodifiedresidue11” (Functional Feature is a modified residue of type “Phosphoserine”, having location 1025). |
| *hasMutation* | Gene | Mutation | EGFR ***hasMutation*** p.L858M (mutation of substitution missense type). |
| *hasNterminus* | Sequence | Nterminus | “Seq-EGFR-UniProt” (EGFR UniProt sequence) ***hasNterminus*** “EGFR-UniProt-Nterminus” (Nterminus having end location 711). |
| *hasParentPathway* | Pathway | Pathway | “EGFR downregulation” ***hasParentPathway*** “Signaling by EGFR”. |
| *hasProteinStructure* | Sequence | Structure | “Seq-EGFR-UniProt” (EGFR UniProt sequence) ***hasProteinStructur****e* “1NQL”. |
| *hasReaction* | Pathway | Reaction | “Signaling by EGFR” ***hasReaction*** *“*EGFR dimerization”. |
| *hasSequence* | Gene | Sequence | EGFR ***hasSequence*** *“*Seq-EGFR-UniProt” (EGFR UniProt sequence). |
| *hasSubDomain* | Sequence | SubDomain | “Seq-EGFR-UniProt” (EGFR UniProt sequence) ***hasSubDomain*** “EGFR-UniProt-G-loop” (G-loop having start location 712 and end location 729). |
| *implicatedIn* | Mutation | Disease | p.L858M (mutation of substitution missense type of EGFR) is ***implicatedIn*** carcinoma. |
| *includes* | Complex, EntitySet | PhysicalEntity | “CDK5:CABLES:ABL [cytosol]” (a complex) ***includes*** ABL1, CDK5 (genes).  “MEK [cytosol]” (an EntitySet) ***includes*** MAP2K1, MAP2K2 (genes). |
| *locatedIn* | Mutation | SubDomain | p.S106N (mutation of substitution missense type of CDK3) is ***locatedIn*** sub domain “CDK3-UniProt-E-helix” (SubDomainV having start location 100 and end location 120). |
| *occursIn* | Mutation | Sequence | p.S106N (mutation of substitution missense type of CDK3) ***occursIn*** “Seq-CDK3-UniProt”. |
| *participatesIn* | Gene | Pathway | EGFR ***participatesIn*** pathway “Signaling by EGFR”. |
| *precededByPathway* | Pathway | Pathway | Pathway “Displacement of DNA glycosylase by APE1” ***precededByPathway*** "Depyrimidination". |
| *precededByReaction* | Reaction | Reaction | “EGFR dimerization” is ***precededByReaction*** “EGFR binds EGF ligand”. |
| *produces* | Reaction | PhysicalEntity | “EGFR dimerization” ***produces*** “EGF:EGFR dimer [plasma membrane]”. |

# 
