# Supplementary material for: ProKinO: An Ontology for Integrative Analysis of Protein Kinases in Cancer
Source: PLoS One. 2011 Dec 14;6(12):e28782. doi: 10.1371/journal.pone.0028782 (PMC3237543; doi:10.1371/journal.pone.0028782)
Supplement: Table S3 — Data properties used in ProKinO. (DOC) [file pone.0028782.s013.doc]

**Table S3.** Data properties used in ProKinO.

| **Data Property** | **Domain Class** | **Example** |
| --- | --- | --- |
| *chromosomalPosition* | Gene | ABL1 has ***chromosomalPosition*** “9q34.2”. |
| *hasCancerType* | Mutation | p.V299L (mutation of substitution missense type of ABL1) ***hasCancerType*** “haematopoietic_neoplasm”. |
| *hasCellularLocation* | Gene | ABL1***hasCellularLocation*** Cytoplasm, Nucleus membrane, Nucleus and cytoskeleton. |
| *hasEndLocation* | FunctionalFeature  SubDomain | “EGFR topological domain 1” (a functional feature topological domain of EGFR) ***hasEndLocation*** “1210”.  “EGFR-UniProt-G-loop” sub-domain ***hasEndLocation*** “729”**.** |
| *hasFastaFormat* | Sequence | “Seq-EGFR-UniProt” (EGFR UniProt sequence) ***hasFastaFormat*** “MRPSGTAGA………………RVAPQSSEFIGA”. |
| *hasFullName* | ProKinOEntity | EGFR ***hasFullName*** “Epidermal growth factor receptor” |
| *hasIdentifier* | DbXref | COSMIC-1006 (an external database cross reference to COSMIC source) ***hasIdentifier*** "NM_004333". |
| *hasIsoformName* | Sequence | Seq-EGFR-Isoform2 (an EGFR isoform) ***hasIsoformName*** “Isoform2”. |
| *hasModifiedResidueType* | ModifiedResidue | *“*EGFR- modifiedresidue11” (a functional feature, namely, modified residue of gene EGFR) ***hasModifiedResidueType*** “Phosphoserine”. |
| *hasMutantType* | Mutation | p.L858M (mutation of substitution missense type of EGFR) ***hasMutantType*** “L”. |
| *hasMutationAA* | Mutation | p.V299L (mutation of substitution missense type of ABL1) ***hasMutationAA*** “p.V299L”. |
| *hasMutationDescription* | Mutation | p.L858M (mutation of substitution missense type of EGFR) ***hasMutationDescription*** “Substitution – Missense”. |
| *hasMutationId* | Mutation | p.L858M (mutation of substitution missense type of EGFR) ***hasMutationId*** “12366”. |
| *hasMutationPosition* | Mutation | p.L858M (mutation of substitution missense type of EGFR) ***hasMutationPosition*** “858”. |
| *hasNameSpace* | DbXref | COSMIC-1006 (an external database cross reference to COSMIC source) ***hasNameSpace*** "http://www.sanger.ac.uk/search?db=cosmic&t=". |
| *hasOtherName* | Gene | EGFR ***hasOtherName*** “EGFRvIII, ERBB1, ERBB, mENA”. |
| *hasPosition* | FunctionalFeature | *“*EGFR- modifiedresidue11” (a functional feature, namely, modified residue of gene EGFR) ***hasPosition*** “1025”. |
| *hasPrimaryName* | ProKinOEntity | “FunctionalDomain-Furin-like” ***hasPrimaryName*** “Furin-like”. |
| *hasPrimarySite* | Mutation | p.L858M (mutation of substitution missense type of EGFR) ***hasPrimarySite*** “lung”. |
| *hasSamplename* | Mutation | p.L858M (mutation of substitution missense type of EGFR) ***hasSamplename*** “83, 982787, 982788”. |
| *hasStartLocation* | FunctionalFeature  SubDomain | “EGFR topological domain 1” (a functional feature topological domain of EGFR) ***hasStartLocation*** “669”.  “EGFR-UniProt-G-loop” sub-domain ***hasStartLocation*** “712”**.** |
| *hasSubDomainSequence* | SubDomain | “EGFR-UniProt-G-loop” sub-domain ***hasSubDomainSequence*** “FKKIKVLGSGAFGTVYKG”. |
| *hasTissueSpecificity* | Gene | ABL1 ***hasTissueSpecificity*** “Widely expressed”. |
| *hasTopologicalDomainType* | TopologicalDomain | “EGFR topological domain 1” (a functional feature topological domain of EGFR) ***hasTopologicalDomainType*** “Cytoplasmic”. |
| *hasURI* | DbXref | COSMIC-1006 (an external database cross reference to COSMIC source) ***hasURI***  "http://www.sanger.ac.uk/search?db=cosmic&t=NM_004333". |
| *hasWildTypeResidue* | Mutation | p.L858M (mutation of substitution missense type of EGFR) ***hasWildTypeResidue*** “M”. |
| *isIsoform* | Sequence | “Seq-EGFR-UniProt” (a EGFR UniProt sequence) ***isIsoform*** “Isoform1”. |
