# Supplementary material for: ProKinO: An Ontology for Integrative Analysis of Protein Kinases in Cancer
Source: PLoS One. 2011 Dec 14;6(12):e28782. doi: 10.1371/journal.pone.0028782 (PMC3237543; doi:10.1371/journal.pone.0028782)
Supplement: Table S4 — ProKinO evaluation statistics. (DOC) [file pone.0028782.s014.doc]

**Table S4.** ProKinO evaluation statistics.

| **Category of Data** | **Data Source** | **Test Data Set** | **Type of Information Evaluated** | **Accuracy** |
| --- | --- | --- | --- | --- |
| Sequence and classification data | KinBase.  The latest release of KinBase out in October 2010 is used to capture the sequence and classification data of kinases in the current version of ProKinO. | A set of protein kinases (60, which is more than 10%) was randomly selected and verified. | The information related to the families, subfamilies, Chromosomal position, other names (synonyms) of protein kinases. | 100% |
| Functional, structure, functional domain and sequence data | UniProt  The current version of ProKinO covers the data from UniProt released on 11^th^ January, 2011. | A set of 5% of all kinases was randomly selected and verified. This verification step was very time consuming, so we limited our verification to 5% of kinases. | The information related to functional domain, structure, functional features (such as, modified residue, topological domain, signal peptide) and comment. | 100% |
| The disease (mutational) data | COSMIC  The current version of ProKinO covers mutational data from the 50^th^ release of COSMIC out on 30^th^ November 2011. | A set of mutated kinases (30, or more than 10% of mutated kinases) was randomly selected and verified. | The information related to Primary Site, Cancer Type, Mutation Id, Mutation AA, Mutation Description, Sample Name, PubMed PMID, Mutation Position, Wild Type, Mutant Type, Mutations of various types (Such as Substitution Missense, Coding Silent, NonSense, Compound Substitution, Complex Insertion in Frame, Complex Deletion in Frame, Deletion In Frame, Deletion, Frameshift Insertion In Frame, Insertion Frameshift, Unknown, and COSMIC Reference (Accession). | 100% |
| Pathway and reaction data | Reactome  The 35th quarterly release of Reactome on 17^th^ December 2010 is covered in the current version of ProKinO for pathway and reaction information. | A set of 5% of all kinases was randomly selected and verified. This verification step was very time consuming, so we limited our verification to 5% of kinases. | The information related to Pathways, Reactions, Other Entities, Complexes, Catalysts, Sub-Pathways, Input to Reactions, Output of Reactions, and Reactome Reference. | 100% |
| Sub-domain data | UniProt, COSMIC, KinBase sequences | A set of mutated kinases (30, or more than 10% of mutated kinases) was randomly selected and verified. | The information related to sub-domain start and end positions, sub-sequence, mutation located in a sub-domain. | 100% |
