# Supplementary material for: ProKinO: An Ontology for Integrative Analysis of Protein Kinases in Cancer
Source: PLoS One. 2011 Dec 14;6(12):e28782. doi: 10.1371/journal.pone.0028782 (PMC3237543; doi:10.1371/journal.pone.0028782)
Supplement: Table S5 — Protein kinases implicated in Haematopoietic and lymphoid_tissue and having modified residue type property. (DOC) [file pone.0028782.s015.doc]

**Table S5.** Protein kinases (top 5) implicated in *Haematopoietic and lymphoid_tissue* and having modified residue type property

| **Protein Kinase** | **ProKinO Id** | **Mutation** | **Mutation Position** | **ProKinO Modified Residue Id** | **Modified Residue Type** | **Modified Residue Position** | **Located In SubDomain** |
| --- | --- | --- | --- | --- | --- | --- | --- |
| **ABL1** | Mutation-12576 | p.Y253H | 253 | ABL1-ModifiedResidue-3 | Phosphotyrosine | 253 | G-loop |
|  | Mutation-12610 | p.Y253F | 253 | ABL1-ModifiedResidue-3 | Phosphotyrosine | 253 | G-loop |
|  | Mutation-12618 | p.Y253H | 253 | ABL1-ModifiedResidue-3 | Phosphotyrosine | 253 | G-loop |
|  | Mutation-12622 | p.Y253F | 253 | ABL1-ModifiedResidue-3 | Phosphotyrosine | 253 | G-loop |
| **ATM** | -- | -- | -- | -- | -- | -- | -- |
| **KIT** | Mutation-24707 | p.Y823D | 823 | KIT-ModifiedResidue-3 | Phosphotyrosine-autocatalysis | 823 | Between Activation-segment-Nt and Activation-segment-Ct |
| **FLT3** | Mutation-19692 | p.Y842C | 842 | FLT3-ModifiedResidue-6 | Phosphotyrosine | 842 | Between Activation-segment-Nt & Activation-segment-Ct |
| **JAK2** | Mutation-33707 | p.Y813D | 813 | JAK2-ModifiedResidue-3 | Phosphotyrosine | 813 | N-terminus |
